# Glutamine Treatment Attenuates Endoplasmic Reticulum Stress and Apoptosis in TNBS-Induced Colitis

**DOI:** 10.1371/journal.pone.0050407

**Published:** 2012-11-28

**Authors:** Irene Crespo, Beatriz San-Miguel, Carolina Prause, Norma Marroni, María J. Cuevas, Javier González-Gallego, María J. Tuñón

**Affiliations:** 1 Centro de Investigación Biomédica en Red de Enfermedades Hepáticas y Digestivas (CIBERehd), León, Spain; 2 Institute of Biomedicine (IBIOMED), University of León, León, Spain; 3 Porto Alegre Clinical Hospital, Federal University of Rio Grande do Sul, Porto Alegre, Rio Grande do Sul, Brazil; Institut Pasteur de Lille, France

## Abstract

Endoplasmic reticulum (ER) stress and apoptotic cell death play an important role in the pathogenesis and perpetuation of inflammatory bowel disease (IBD). We aimed to explore the potential of glutamine to reduce ER stress and apoptosis in a rat model of experimental IBD. Colitis was induced in male Wistar rats by intracolonic administration of 30 mg of 2,4,6-trinitrobenzene sulfonic acid (TNBS). Glutamine (25 mg/dL) was given by rectal route daily for 2 d or 7 d. Both oxidative stress (TBARS concentration and oxidised/reduced glutathione ratio) and ER stress markers (CHOP, BiP, calpain-1 and caspase-12 expression) increased significantly within 48 h of TNBS instillation, and glutamine attenuated the extent of the changes. Glutamine also inhibited the significant increases of ATF6, ATF4 and spliced XBP-1 mRNA levels induced by TNBS instillation. TNBS-colitis resulted in a significant increase in p53 and cytochrome c expression, and a reduced Bcl-xL expression and Bax/Bcl-2 ratio. These effects were significantly inhibited by glutamine. Treatment with the amino acid also resulted in significant decreases of caspase-9, caspase-8 and caspase-3 activities. Double immunofluorescence staining showed co-localization of CHOP and cleaved caspase-3 in colon sections. Phospho-JNK and PARP-1 expression was also significantly higher in TNBS-treated rats, and treatment with glutamine significantly decreased JNK phosphorylation and PARP-1 proteolysis. To directly address the effect of glutamine on ER stress and apoptosis in epithelial cells, the ER stress inducers brefeldin A and tunicamycin were added to Caco-2 cells that were treated with glutamine (5 mM and 10 mM). The significant enhancement in PERK, ATF6 phosphorylated IRE1, BiP and cleaved caspase-3 expression induced by brefeldin A and tunicamycin was partly prevented by glutamine. Data obtained indicated that modulation of ER stress signalling and anti-apoptotic effects contribute to protection by glutamine against damage in TNBS-induced colitis.

## Introduction

Inflammatory bowel disease (IBD), which two major forms are Ulcerative colitis (UC) and Crohn’s disease (CD), is a multifactorial inflammatory disease of the colon and rectum. Although its aethiology remains unknown, many factors, such as neutrophil infiltration and overproduction of proinflammatory mediators, including cytokines and reactive oxygen mediators, are implicated in the pathogenesis of IBD [1], [2]. Previous reports suggest that the endoplasmic reticulum (ER) stress response is also induced in association with the development of IBD [3], [4]. However, its contribution to the pathogenesis of IBD remains unclear. The physiological role of the ER includes the synthesis, folding and modification of secretory and transmembrane proteins [5]. Any disturbance of ER homeostasis can result in excessive accumulation of misfolded or unfolded proteins in the ER lumen. This accumulation leads to ER stress, and triggers the unfolded protein response (UPR), which initiates the development of apoptosis [6]. Three proximal effectors of the UPR exist in mammalian: pancreatic ER kinase (PERK), activating transcription factor 6 (ATF6), and inositol-requiring transmembrane kinase/endonuclease 1 (IRE1). Autophosphorylation of PERK permits the translation of specific cap-independent ER stress response genes, such as ATF4. Secondly, ATF6 is activated by proteolytic cleavage following traslocation to the Golgi. Finally, IRE1 autophosphorylation promotes the splicing of X-box-binding protein-1 (XBP-1) mRNA to its short form XBP-1s [7]. Association of the immunoglobulin-heavy-chain-binding protein (BiP/GRP78) fine-tunes IRE1 signaling, while unfolded proteins act as activating ligands of ER stress sensors [8]. Epithelial-specific deletion of XBP-1 in mice results in spontaneous ileitis and increased susceptibility to chemically induced colitis [9], and activation of the three UPR-related arms has been recently reported in colonic IBD [10]. ER stress also induces phosphorylation of c-Jun N-terminal kinase (JNK) [11], a family member of the stress-activated protein kinases, whose activation has been proposed to be a proapoptotic event through direct phosphorylation of mitochondrial proteins, including members of the B-cell lymphoma (Bcl-2) family of proteins [12]. Although many factors are involved in the apoptotic program, caspases have been shown to play a major role in the transduction of apoptotic signals and several studies have demonstrated that ER stress induces activation of caspases [13], [14]. Apoptosis increases in gastrointestinal diseases such as colon and pancreas cancer, acute pancreatitis, and radiation enteritis [15]. The frequency of apoptosis and its contribution to the loss of epithelial cells is also considerably increased in IBD [12].

Conventional IBD therapy typically involves pharmacological agents such as aminosalicylates, corticosteroids and immunosuppressive drugs. However, these treatments have demonstrated variable efficacy, adverse side effects and potential long-term toxicity [16]. Therefore, the need for alternative therapeutic strategies is of utmost importance. Amino acids are key regulators of metabolic pathways, and evidence has indicated additional roles for amino acids in maintaining gut health [17]. Glutamine, the most abundant amino acid in the bloodstream, plays a central role in nitrogen transport within the body, is a fuel for rapidly-dividing cells, and has many other essential metabolic functions. Lower levels of glutamine have been associated with immune dysfunction and increased mortality [18], and it has been reported that glutamine therapy improves outcome of *in vitro* and *in vivo* experimental colitis models [19]. A mechanism by which glutamine seems to exert its beneficial effects appear to be correlated with the decrease of oxidative stress [19], [20]. In addition, different studies have shown that glutamine supplementation delays human neutrophil apoptosis and reduced T-cell apoptosis [21]. Glutamine deprivation also induces apoptosis in rat intestinal epithelial cells [22] and renders premonocytic and HL-60 cells significantly more susceptible to Fas-mediated apoptosis [23].

In previous research we have demonstrated that treatment with glutamine markedly decreases the severity of macroscopic damage and the histopathological scores in several experimental animal models of colitis [1], [2]. Reduced myeloperoxidase activity and expression of inducible nitric oxide synthase, cyclooxygenase-2 and adhesion molecules confirmed the anti-inflammatory effect of glutamine. These protective effects are associated with changes in nuclear factor kappa B and signal transducers and activators of transcription (STAT) signaling pathways [1], [2]. Moreover, glutamine treatment not only attenuates the outcome of colitis by impairing the inflammatory response, but also by reducing the risk of fibrosis and stricture formation through down-regulation of several gene pathways that contribute to the accumulation of matrix proteins [24]. The purpose of our study was to investigate, using both *in vitro* and *in vivo* models, whether inhibition of ER stress and apoptosis contributes to the beneficial effects of glutamine. The present research provides evidence that reduction of colon damage by glutamine is associated with direct attenuation of ER stress through a modulation of the three arms of UPR signaling, and with a diminution of apoptotic cell death.

## Results

### Glutamine Inhibits Oxidative Stress in Rats with TNBS-induced Colitis

Oxidative stress is an important contributor to the pathogenesis of IBD [25]. The presence of oxidative stress was determined by measurement of the cytosolic concentration of thioarbituric acid reactive substances (TBARS) and the oxidised/reduced (GSSG/GSH) ratio. Data shown in Table 1 indicate that TBARS concentration increased in colonic samples taken from rats receiving TNBS at different time points. Treatment with 25 mg/kg of glutamine attenuated this effect. GSSG/GSH ratio was also significantly higher in rats with experimental colitis. Glutamine prevented this elevation and values did not significantly differ from untreated controls (Table 1).

**Table 1:** Effect of treatment with glutamine on markers of oxidative stress in rats with TNBS-induced colitis at 2 d and 7 d of saline or TNBS instillation.

	Groups					
	Control	Control+G	TNBS 2d	TNBS+G 2d	TNBS 7d	TNBS+G 7d
TBARS (nmol/mg protein)	0.96±0.04	0.93±0.029	2.14±0.06*	1.22±0.05* #	3.88±0.05* &	1.65±0.044* #
GSH (µmol/mg protein)	3.42±0.29	3.10±0.13	2.32±0.21	2.57±0.15	2.36±0.15	3.45±0.11&
GSSG (µmol/mg protein)	0.11±0.01	0.10±0.01	0.11±0.01	0.08±0.01* #	0.11±0.01	0.10±0.01
(GSSG/GSH)x100	3.20±0.11	3.22±0.09	4.74±0.19*	2.92±0.12#	4.66±0.17*	2.87±0.09#

Data are expressed as mean ± S.E.M. of 8 rats.

* *P<0.05* compared with control group.

# *P<0.05* compared with TNBS group.

& *P<0.05* compared with same group 2 d.

### Glutamine Reduces ER Stress in Rats with TNBS-induced Colitis

ER stress represents a new pathway that involves the intestinal epithelium, and several reports suggest that the IBD is associated with an induction of the ER stress [3], [4], [10]. Taken together, the active transcription factors ATF6, ATF4, and spliced XBP-1 (XBP-1s) regulate the expression of ER chaperones that enhance the folding capacity of the ER, including BiP, as well as other stress genes such as CCAAT/enhancer-binding protein homologous protein (CHOP) [26]. In our study, mRNA expression level of XBP-1s increased only at 7 d of treatment with TNBS. However, the induction of colitis by TNBS resulted in significant increases in the mRNA levels of ATF6, ATF4, BiP and CHOP, both at 2 d and 7 d. Values were significantly lower in the rats which received TNBS plus glutamine (Table 2).

**Table 2:** Effect of treatment with glutamine on mRNA levels of genes related to ER stress in rats with TNBS-induced colitis at 2 d and 7 d of saline or TNBS instillation.

	Fold of control					
	Control	Control+G	TNBS 2d	TNBS+G 2d	TNBS 7d	TNBS+G 7d
ATF4	1.00±0.02	1.09±0.07	1.58±0.14*	0.92±0.08#	1.41±0.10*	0.98±0.02#
ATF6	1.00±0.07	1.11±0.04	1.66±0.10*	1.28±0.04* #	1.81±0.19*	0.98±0.10#
XBP-1s	1.00±0.08	0.99±0.05	1.09±0.02	1.10±0.15	1.42±0.05* &	1.13±0.06#
CHOP	1.00±0.09	1.03±0.03	1.48±0.13*	0.91±0.07#	1.70±0.16*	1.13±0.04#
BiP	1.00±0.04	1.12±0.06	1.54±0.08*	1.36±0.12*	1.30±0.06*	1.12±0.09^#&^

Data are expressed as mean ± S.E.M. of 8 rats.

* *P<0.05* compared with control group.

# *P<0.05* compared with TNBS group.

& *P<0.05* compared with same group 2 d.

**Figure 1:**
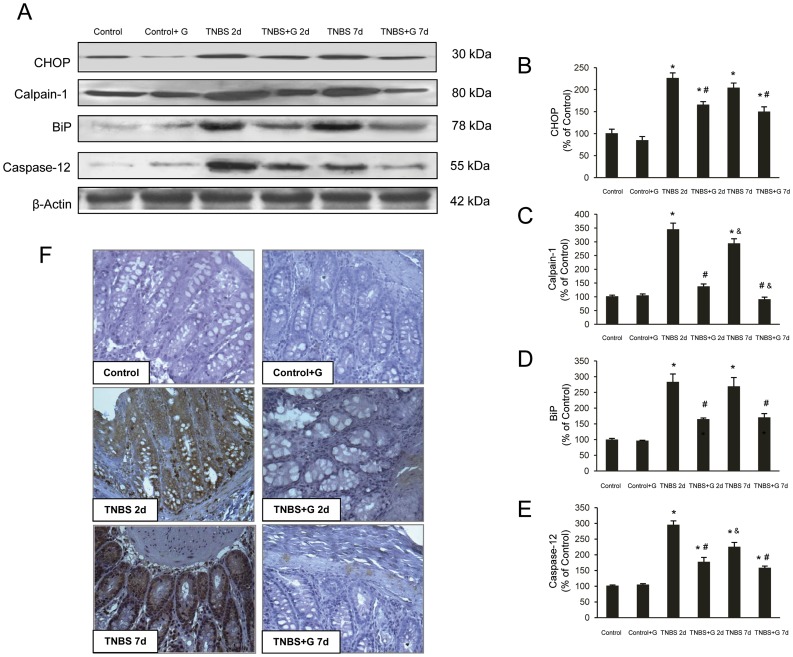
Glutamine reduces the ER stress induced by TNBS-colitis. (A–E). Protein from colonic extracts was separated by sodium dodecyl sulfate-polyacrylamide gel electrophoresis, followed by immunoblotting for CHOP, BiP, calpain-1 and caspase-12. CHOP, BiP, calpain-1 and caspase-12 were markedly expressed in rats treated with TNBS alone. However, glutamine administration partially abolished CHOP, BiP, calpain-1 and caspase-12 expression induced by TNBS. Results are representative of four independent experiments. Equal loading of proteins is illustrated by β-actin bands. (A) Representative Western-blot photographs for CHOP, calpain-1, BiP, caspase-12, and β-actin. (B) Densitometric quantification of CHOP. (C) Densitometric quantification of calpain-1. (D) Densitometric quantification of BiP. (E) Densitometric quantification of caspase-12. Data are expressed as mean ± S.E.M. from 8 rats. *P<0.05 compared with control group. ^#^P<0.05 compared with TNBS group. ^&^P<0.05 compared with same group 2 d. (F) Photomicrographs of immunohistochemistry for BiP in sections of colonic samples. Paraffin-embedded sections were immunostained with a BiP antibody. Original magnification: 200X.

We also investigated by Western blot CHOP, BiP, calpain-1 and caspase-12 to identify effects of glutamine on the activation of factors involved in the ER stress of TNBS-inflamed colon tissues. Fig. 1 shows that at 2 d and 7 d after treatment, exposure to TNBS caused higher expression of CHOP, BiP, calpain-1 and caspase-12 in extracts from colonic mucosa. Protein levels were significantly lower in glutamine-treated rats receiving TNBS (Fig. 1A–E).

In order to confirm if ER-stress was increased in epithelial cells, immunohistochemistry for BiP, a major marker of the ER stress response, was performed. Immunoreactivity for BiP was negative in colon section from control rats. In comparison to the TNBS-treated groups, immunoreactivity was markedly reduced in rats with colitis receiving glutamine (Fig. 1F).

### Glutamine Reduces Apoptosis in Rats with TNBS-induced Colitis

**Figure 2:**
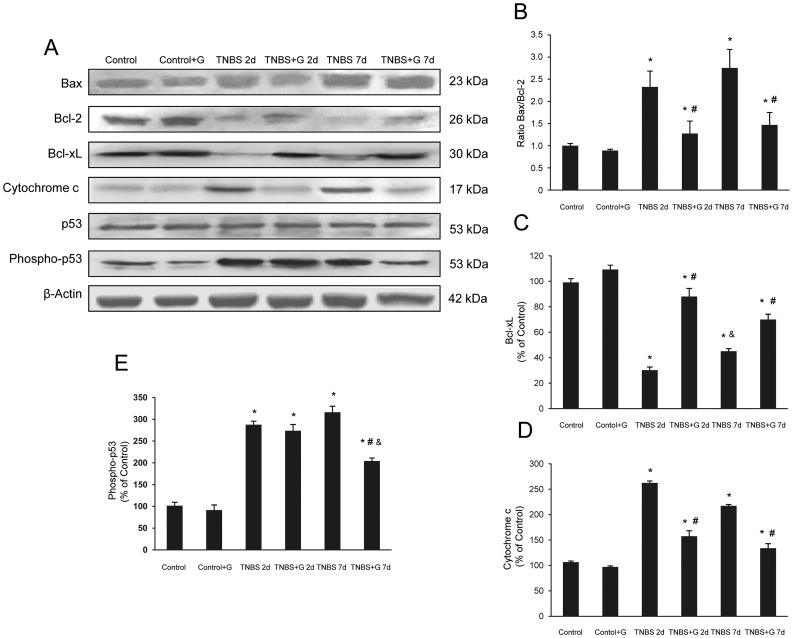
Glutamine reduces the apoptotic pathways induced by TNBS-colitis. (A–E) Protein from colonic extracts was separated by sodium dodecyl sulfate-polyacrylamide gel electrophoresis, followed by immunoblotting for Bax, Bcl-2, Bcl-xL, cytochrome c, p53 and phospho-p53. Bax, cytochrome c and phospho-p53 were markedly expressed in rats treated with TNBS alone. On the contrary, Bcl-2 and Bcl-xL were markedly reduced in rats treated with TNBS alone. However, glutamine administration partially abolished the changes in Bax, Bcl-2, Bcl-xL, cytochrome c and phospho-p53 expression induced by TNBS. Results are representative of four independent experiments. Equal loading of proteins is illustrated by β -actin bands. (A) Representative Western-blot photographs for Bax, Bcl-2, Bcl-xL, cytochrome c, p53, phospho-p53, and β-actin. (B) Densitometric quantification of ratio Bax/Bcl-2. (C) Densitometric quantification of Bcl-xL. (D) Densitometric quantification of cytochrome c. (E) Densitometric quantification of phospho-p53. Data are expressed as mean ± S.E.M. from 8 rats. *P<0.05 compared with control group. ^#^P<0.05 compared with TNBS group. ^&^P<0.05 compared with same group 2 d.

In IBD, frequency of apoptosis is considerably increased and loss of epithelial cells appears to occur mainly by apoptosis [27]. To identify the apoptotic pathways inhibited by glutamine, we examined different markers of apoptosis. The expression of the pro-apoptotic protein phospho-p53 showed a significant increase in the group receiving TNBS when compared with control rats. Glutamine partially prevented this effect after 7 d of treatment (Fig. 2A, E). Bax is a member of the Bcl-2 family that also favours apoptosis, contributing to the release of the intermembrane mitochondrial cytochrome c. Fig. 2 shows a slight increase of Bax and a significantly increased expression of cytosolic cytochrome c in colon of rats receiving TNBS, which were prevented by glutamine (Fig. 2A, D). Formation by Bax of the mitochondrial pore that allows the release of cytochrome c is prevented by Bcl-2. Expression of this antiapoptotic protein was markedly impaired in TNBS-inflamed colon tissues, but increased in rats receiving glutamine (Fig. 2A). When the Bax/Bcl-2 ratio was calculated according to Western blotting results, values were significantly lower in TNBS plus glutamine compared to TNBS both at 2 d and 7 d of instillation (Fig. 2B). Inhibition of the expression of Bcl-xL, another antiapoptotic protein of the Bcl-2 family, was also significantly prevented by glutamine (Fig. 2A, C).

**Figure 3:**
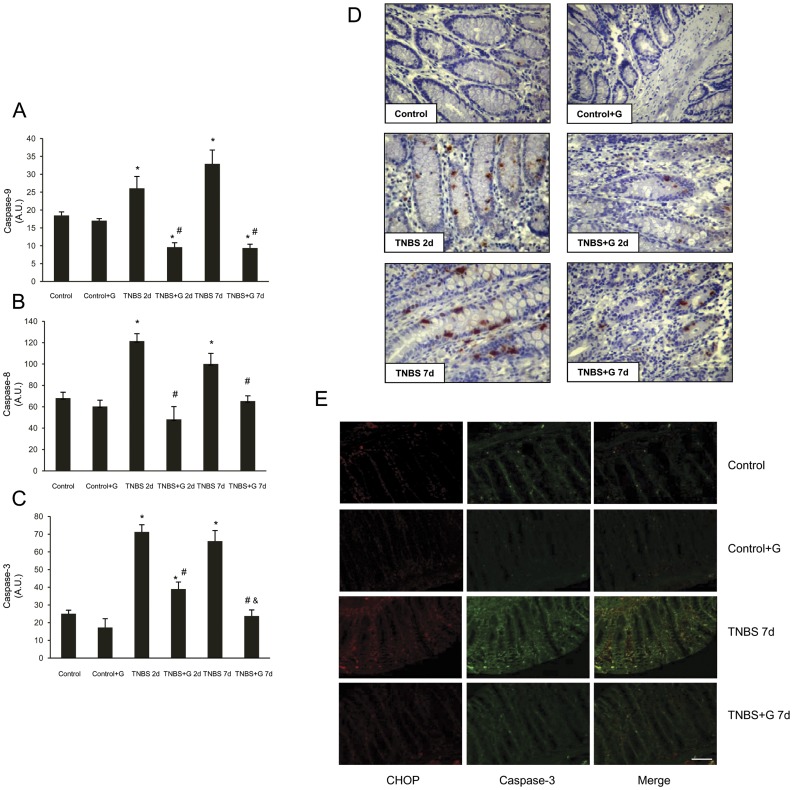
Glutamine reduces the caspase activities induced by TNBS-colitis. (A–C) Colon activity of caspase-9, caspase-8, and caspase-3 were markedly increased in rats treated with TNBS alone. However, glutamine administration abolished caspase-9, caspase-8, and caspase-3 activities induced by TNBS. (A) Activity of caspase-9. (B) Activity of caspase-8. (C) Activity of caspase-3. Data are expressed as mean ± S.E.M. from 8 rats. *P<0.05 compared with control group. ^#^P<0.05 compared with TNBS group. ^&^P<0.05 compared with same group 2 d. (D) Photomicrographs of immunohistochemistry for cleaved caspase-3 in sections of colonic samples. Paraffin-embedded sections were immunostained with a cleaved caspase-3 antibody. Original magnification: 200X. (E) Photomicrographs of double immunofluorescence for CHOP and cleaved caspase-3 in sections of colonic samples. Paraffin-embedded sections were double staining with a CHOP (red) and cleaved caspase-3 (green) antibodies, and the yellow colour visualized in the merged images represented co-localization of CHOP with cleaved caspase-3. Data shown are representative from four rats. Scale bar 50 µm.

To determine whether caspases were activated by hapten-induced colitis, samples were incubated with specific fluorigenic substrates, whose cleavage indicated that exposure to TNBS resulted in marked increases in caspase-9, caspase-8, and downstream caspase-3 activities. These effects were prevented by glutamine (Fig. 3A–C). To further assess activation, immunohistochemistry for caspase-3 was performed. No positively stained cells appeared in control rats. In colon sections from rats receiving glutamine, the number of positively stained cells was markedly reduced in comparison to those detected in TNBS-induced colitis (Fig. 3D).

A double immunofluorescence analysis for CHOP and for cleaved caspase-3 was performed in colon sections to confirm the correlation between the levels of ER stress and the apoptotic cell death. TNBS induced at 7 d expression of CHOP and cleaved caspase-3, compared to the control group (Fig. 3E). Double staining also showed that co-localization of both proteins in colon sections. Immunostaining decreased markedly with glutamine treatment.

### Glutamine Reduces JNK Phosphorylation and Poly (ADP-ribose) Polymerase (PARP)-1 Expression in Rats with TNBS-induced Colitis

**Figure 4:**
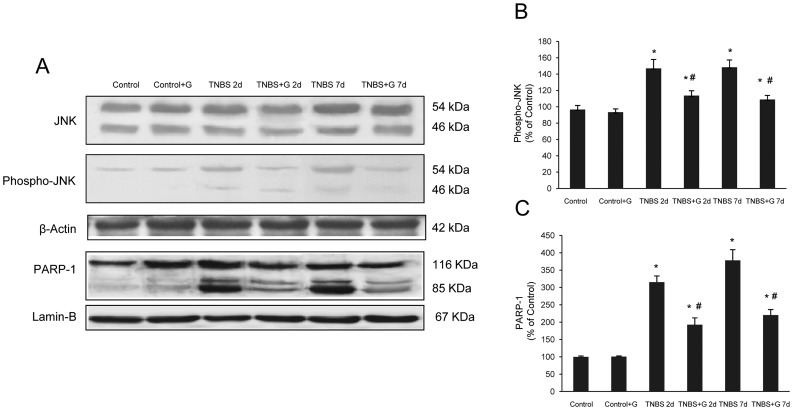
Glutamine reduces the JNK phosphorylation and PARP-1 proteolysis induced by TNBS-colitis. (A–C) Protein from colonic extracts was separated by sodium dodecyl sulphate-polyacrylamide gel electrophoresis, followed by immunoblotting for JNK, phospho-JNK (cytosolic extracts) and PARP-1 (nuclear extracts). Phospho-JNK and PARP-1 were markedly expressed in rats treated with TNBS alone. However, glutamine administration partially abolished phospho-JNK and PARP-1 expression induced by TNBS. Results are representative of four independent experiments. Equal loading of proteins is illustrated by β-actin (cytosolic extracts) and lamin B (nuclear extracts) bands. (A) Representative Western-blot photographs for JNK, phospho-JNK, β-actin, PARP-1 and lamin-B. (B) Densitometric quantification of phospho-JNK. (C) Densitometric quantification of PARP-1. Data are expressed as mean ± S.E.M. from 8 rats. *P<0.05 compared with control group. ^#^P<0.05 compared with TNBS group.

JNK phosphorylation is secondary to ER stress and may participate in the development of apoptosis [11]. We further determined the expression of the active phosphorylated form of JNK by Western blot. Analysis showed that JNK activity steadily increased at 2 d after TNBS administration and remained activated at 7 d. Administration of glutamine to TNBS-instillated rats resulted in a significant loss of JNK phosphorylation (Fig. 4A, B).

Inhibition of the nuclear enzyme PARP-1 may reduce the apoptotic process by shifting the ratio of apoptotic regulators along with reduction of JNK activity, and beneficial effects of inhibitors of this nuclear polymerase have been reported in experimental models of colitis [12], [28]. We compared the expression of PARP-1 in rats subjected to TNBS-induced colitis and colitic rats treated with glutamine. Western blot analysis demonstrated a marked PARP-1 proteolysis in rats with experimental colitis, which was significantly lower in the groups of TNBS-treated rats which received glutamine (Fig. 4A, C).

### Glutamine Reduces ER-stress and Apoptosis in Caco-2 Cells

The direct anti-ER stress effect of glutamine was established in *in vitro* experiments using brefeldin A or tunicamycin as ER stressors. Caco-2 cells were differentiated after 2 weeks of incubation and assumed intestinal epithelium like features. After cells were cultured with brefeldin A or tunicamycin and treated with glutamine 5 mM or 10 mM cell viability was measured by 3-(4,5-dimethylthiazol-2-yl)-2,5-diphenyltetrazolium bromide (MTT) assay. Incubation with the ER stressors and glutamine treatment did not induce any significant change in cell viability (data not shown).

**Figure 5:**
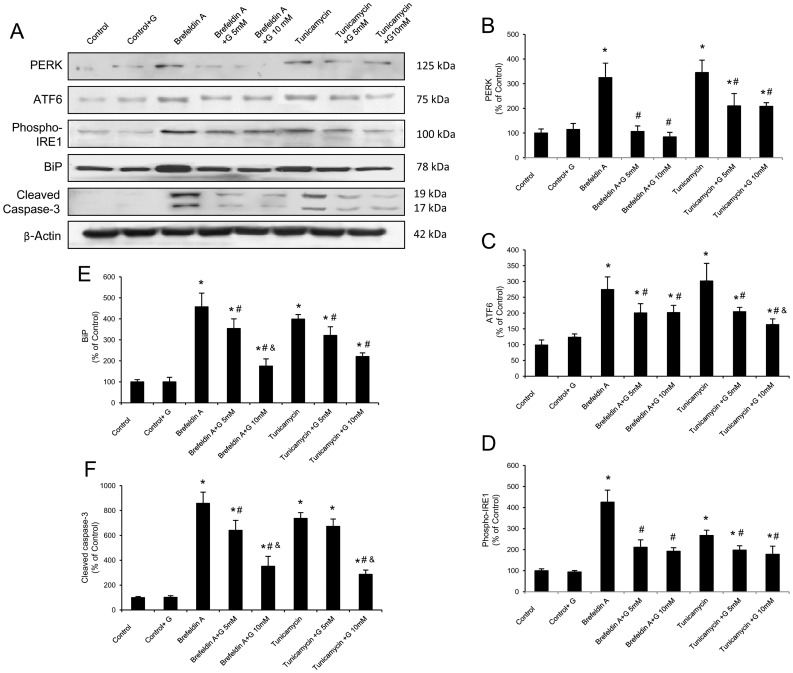
Glutamine reduces the ER stress and apoptosis induced by brefeldin A and tunicamycin in Caco-2 cells. (A–F) Protein from Caco-2 cells was separated by sodium dodecyl sulfate-polyacrylamide gel electrophoresis, followed by immunoblotting for PERK, ATF6, phosphorylated IRE1 (phospho-IRE1), BiP and cleaved caspase-3. PERK, ATF6 phospho-IRE1, BiP and cleaved caspase-3 were markedly expressed in cells treated with brefeldin A or tunicamycin alone. However, glutamine administration (5 and 10 mM) partially abolished the changes induced by brefeldin A and tunicamycin. Results are representative of four independent experiments. Equal loading of proteins is illustrated by β-actin bands. (A) Representative Western-blot photographs for PERK, ATF6, phospho-IRE1, BiP, cleaved caspase-3 and β-actin. (B) Densitometric quantification of PERK. (C) Densitometric quantification of ATF6. (D) Densitometric quantification of phospho-IRE1. (E) Densitometric quantification of BiP. (F) Densitometric quantification of cleaved caspase-3. Data are expressed as mean ± S.E.M. *P<0.05 compared with control group. ^#^P<0.05 compared with same stress inducer without glutamine group. ^&^P<0.05 compared with same stress inducer +5 mM glutamine group.

Brefeldin A and tunicamycin treatment induced a significant up-regulation of BiP. When Caco-2 cells were treated with glutamine in combination with brefeldin A or tunicamycin, glutamine showed an inhibitory effect on BiP protein concentration (Fig. 5A, E). Moreover, in cells treated with the two ER stressors, glutamine reduced the expression of the ER stress sensors PERK, ATF6 and phosphorylated IRE1 (Fig. 5A, B–D). Data obtained demonstrate that all UPR signaling branches are inhibited *in vitro* by glutamine administration.

To investigate the effect of glutamine in the relationship between ER stress and apoptosis, cleaved caspase-3 was also determined in Caco-2 cells. An induction at the active caspase-3 protein level was observed after treatment with brefeldin A and tunicamycin. Glutamine administration resulted in a significant inhibitory effect on caspase-3 induction by both ER stressors (Fig. 5A, F).

## Discussion

Alteration of epithelial function is associated in IBD with an aberrant production of reactive oxygen and nitrogen species. Several studies suggest that peripheral blood monocytes and isolated intestinal macrophages from patients with IBD produce increased amounts of free radicals [29]. High numbers of peripheral neutrophils, which are capable of generating large amounts of oxygen-derived free radicals, also migrate into the intestinal wall of IBD patients [30]. Evidence consistent with damage by reactive radical species is also provided by the increase in lipid peroxides in rectal biopsy specimens from patients with UC [31]. Data in the present study confirm these findings by showing that both the colonic concentration of TBARS and the molecular ratio between GSSG and GSH, sensitive indicators of the cellular redox state, were significantly increased within 2 d of TNBS instillation. In the TNBS model of IBD it has been shown that prophylactic glutamine administration is associated with decreased TBARS and increased glutathione levels in colonic mucosa [2]. A mechanism by which glutamine seems to exert its beneficial effects appear to be correlated with the biosynthesis of glutathione, since is the precursor of the glutamate used for glutathione synthesis. The present finding that glutamine prevents increases in TBARS concentration and GSSG/GSH ratio after both 2 d and 7 d of TNBS instillation supports that inhibition of oxidative stress contributes to the attenuation of colonic damage by glutamine.

In the reducing environment of the mammalian cytosol, GSH exceeds GSSG by a ratio between 30∶1 and 100∶1. A significant increase of this ratio is reflective of a more oxidizing compartment, and alterations of the luminal redox conditions affect protein processing, are sensed by the accumulation of misfolded/unfolded proteins, and may induce ER stress and unfolded protein response [32]. The activated signaling pathways attempt to restore the balance between protein loading and processing and induce programmed cell death if these attempts fail. Recent findings strongly support the involvement of redox-based ER stress in a plethora of human diseases, including IBD, either as causative agents or as complications [33]. The transcription factor CHOP is a good marker of ER stress, because it is expressed specifically under the conditions of ER dysfunction [6]. It has been shown that CHOP is up-regulated by dextran sulfate sodium (DSS) or TNBS administration and that CHOP-null mice are resistant to development of experimental colitis in these models [29]. In addition to CHOP, other molecules may be involved in the ER-dependent exacerbation of TNBS-induced colitis. Thus, the chaperone BiP has been recently shown to play a central role modulating the sensitivity and duration of the UPR [8]. Consistent with the notion that intestinal oxidative stress/inflammation can secondarily induce ER stress in IBD is the observation that BiP expression is increased to a similar degree in epithelial cells from patients with CD, UC as well non-IBD inflammatory controls (sigmoid diverticulitis) compared to uninflamed controls [34]. Our results support this idea, and up-regulation of CHOP and BiP is observed in the TNBS-inflamed colon. On the other hand, down-regulation of CHOP activity compromises cell viability, and cells lacking CHOP are significantly protected from the lethal ER stress [35]. Glutamine treatment reduces CHOP and BiP expression in TNBS rats and may therefore be involved in the inhibition of both the initiation and/or perpetuation of mucosal inflammation in IBD. However, the decrease in ER stress after treatment with glutamine is not simply due to diminished inflammation and is not indicative of the level of inflammation and/or oxidative stress. In fact, results from our additional *in vitro* studies in Caco-2 cells treated with the ER stressors brefeldin A and tunicamycin demonstrated that glutamine may directly attenuate ER stress in epithelial cells and alleviate UPR signaling emerging from diverse types of ER insults. Thus, protein concentration of BiP and CHOP was reduced by glutamine in cells treated with brefeldin A, which disrupts ER-to-Golgi vesicle trafficking, and also in cells treated with tunicamycin, an inhibitor of N-linked glycosylation, which constitutes an early event in protein folding within the ER [36].

To better characterize the effect of glutamine in TNBS-induced stress signaling, we investigated mRNA levels of factors involved in the three individual UPR signaling branches. Data obtained indicate that TNBS-instillation resulted in significant increases of ATF4 and ATF6 mRNA levels both a 2 d and 7 d, while splicing of XBP-1 mRNA was enhanced at 7 d. Our results confirm previous research indicating that activation of the ATF6, IRE and PERK signaling branches is seen in patients with colonic IBD [10], and are also consistent with data from mice with conditional deletion of XBP-1 in the intestinal epithelium [9]. Data obtained also demonstrate that all UPR signaling branches are inhibited in TNBS-treated rats by glutamine administration. This was further confirmed by the fact that in Caco-2 cells treated with the two ER stressors, glutamine reduced the expression of the ER stress sensors PERK, ATF6 and phospho-IRE1.

At the final step of mammalian ER stress response, the apoptotic response is initiated to eliminate cells. CHOP is involved in ER stress-induced apoptosis through various mechanisms such as down-regulation of Bcl-2 and translocation of Bax to mitochondria [29]. BiP has also demonstrated its role in ER stress-mediated apoptosis both in *in vivo* and *in vitro* studies [37], [38]. Caspase-12, a murine protein also associated with the ER membrane, normally exists in an inactive procaspase form. During ER stress, caspase-12 dissociates from the ER membrane is cleaved to a fragment, and then activates, initiating downstream apoptotic pathways. Caspase-12-deficient mice are resistant to ER stress-induced apoptosis [39]. Calpains have been proposed to mediate processing and activation of caspase-12 after induction of the unfolded protein response and ER stress [40]. Calpain-deficient mouse embryonic fibroblasts show decreased ER stress-induced activation of caspase-12 and are resistant to ER stress-induced apoptosis [41]. Our data indicate that both caspase-12 and calpain-1 are significantly induced in the colonic mucosa of TNBS-treated rats and this induction is partly abolished by glutamine.

Reactive oxygen and nitrogen species formation and ER stress may result in the expression of genes for pro-inflammatory mediators and cellular death by apoptosis [28]. In UC, the frequency of apoptosis is considerably increased and loss of epithelial cells appears to occur mainly by apoptosis. In fact, previous research has shown significant apoptosis in colonic epithelial cells during mild acute inflammation induced by DSS [42] and TNBS-induced colitis [43]. In our study, a significant increase in the pro-apoptotic Bax protein was found in the colon tissue of TNBS-treated rats. In addition, the expression of the anti-apoptotic Bcl-2 and Bcl-xL proteins significantly decreased in the TNBS group. Bcl-2 and Bcl-xL function to prevent cell death, whereas Bax accelerates the cell death signal [44]. Because the ratio of Bax/Bcl-2, a parameter of apoptotic cell death, was increased in colon tissue of TNBS treated rats, it appears that apoptosis was involved in TNBS-induced colitis. The present findings are in accordance with other studies, in which colonic cell death was associated with apoptosis in the colon lesion 48 h after intracolonic administration of TNBS [27]. Our results also indicate that glutamine induces a slight non significant decrease in Bax protein level that is accompanied by increased Bcl-2 and Bcl-xL. Furthermore, the relative Bax/Bcl-2 ratio decreased with glutamine treatment, thereby skewing the balance away from one which would favor cell survival as seen in the control rats, even at early time points. Finally, although many factors are involved in the apoptotic program, caspases have been shown to play a major role in the transduction of apoptotic signals. In line with this, caspase-3, caspase-9 and caspase-8 activities of colonic tissues was significantly higher in TNBS-treated rats compared to the control group, while treatment with glutamine significantly decreased caspase activities compared to that in TNBS-treated rats. The relationship of ER stress and apoptosis was supported by results from the double immunofluorescence analysis for CHOP and cleaved caspase-3, and by data obtained in Caco-2 cells experiments indicating that BiP and cleaved caspase-3 expression were reduced by glutamine in cells treated with brefeldin A and tunicamycin.

The present results also demonstrate that the ER stress-related proteins, CHOP, BiP and caspase-12, increase concomitantly with phospho-JNK. During inflammation, oxidative and nitrosative stress represents an important signal for the activation of JNK. Moreover, JNK is also activated by the ER stress in mammalian cells [11]. JNK activation has been proposed to be a proapoptotic event through direct phosphorylation of mitochondrial proteins, including Bcl-2 family members [12]. Therefore, it is possible that the elevated and steady activity of JNK may be required for the activation of different signaling cascades, including apoptotic pathways at different stages of the inflammatory process. Furthermore, it is known that inhibition of the nuclear enzyme PARP-1 may reduce the apoptotic process by shifting the ratio of apoptotic regulators towards Bcl-2, along with reduction of JNK activity [12], [28]. In fact, excessive activation of PARP-1 leads to the loss of cell membrane integrity and viability. On the contrary, inhibition of poly(ADP-ribosyl)ation preserves the cellular energy pool, thus preventing metabolic failure and providing cytoprotection, and previous *in vivo* studies demonstrated that genetic ablation of PARP-1 ameliorates the pathophysiological changes of experimental colitis [12]. Inhibition of colon damage by glutamine was associated with a significant reduction of the activation of JNK and reduction of PARP-1 expression. These data support a pathological role of PARP-1 in colitis, possibly by regulating the early stress-related transcriptional response through a positive modulation of the JNK pathways.

In summary, the present study supports the idea that treatment with glutamine attenuates the outcome of TNBS-induced colitis and reinforces the usefulness of exploring glutamine as a potential alternative therapeutic strategy in IBD. Protection associated with glutamine is due not only to the previously reported anti-inflammatory effects of this amino acid, but also to a modulation of ER stress signaling and a prevention of apoptosis development. Our data suggest that the inhibition of different mechanisms, such as factors involved in the ER stress response (CHOP, BiP and caspase-12), UPR signaling branches (ATF6, ATF4, XBP-1s), the mitogen-activated protein kinase JNK, Bcl-2 family proteins, and caspase activation, would be implicated in the beneficial effects of glutamine in experimental colitis.

## Materials and Methods

### Ethics Statement

This study was carried out in strict accordance with the recommendations in the Guide for the Care and Use of Laboratory Animals of the National Institutes of Health. The study was specifically approved by the Ethics Committee of the University of León (Permit Number: LE026A08). All surgery was performed under isoflurane anesthesia, and all efforts were made to minimize suffering.

### Rats and Induction of Colitis

Male Wistar rats weighing 300–350 g, provided by Panlab (Barcelona, Spain), were caged at 24°C, with a 12 h light dark cycle and free access to standard food and water until the time of experiments. Experimental colitis was induced by TNBS according to the procedure described by Morris et al. [45]. Briefly, rats fasted for 24 h were lightly anesthetized with isoflurane, and a polyethylene catheter (2 mm in outer diameter) was inserted rectally until the splenic flexure (6–8 cm from the anus). 30 mg of TNBS (Sigma, St Louis, USA) dissolved in a volume of 0.25 mL of ethanol 50% (v/v) were administered through the catheter. TNBS was retained in the colon for 30 s, after which the fluid was withdrawn.

### Rats and Treatments

The rats were randomly divided into 4 groups up to 10 animals: a colitis group which received TNBS, a control group which received only vehicle, and two additional groups which received by rectal route glutamine (G) (Sigma) (25 mg/kg/day in a volume of 3 mL of 0.9% saline), 4 h after the induction of colitis and once daily up to the end of the study at d 7 [46]. Since molecular alterations likely precede clinical signs of colitis and histopathological evidence of inflammation, signs of apoptosis and other molecular events were evaluated at earlier time points. Thus, the same experimental design was repeated and additional groups of rats were sacrificed at 48 h of vehicle or TNBS instillation. The rats were killed, and the distal 8 cm of the colon was excised, opened by longitudinal incision, rinsed with saline, immediately snap-frozen in liquid nitrogen, and stored at −80°C.

### Cell Culture

In a set of confirmatory experiments, the human colon cancer cell lines Caco-2 from the European Collection of Cell Cultures were routinely grown. Caco-2 cells (passages 45–50) were seeded at the density of 1.2×10^5^ cell.cm^−2^ onto 60 mm plastic dishes (Corning, NY, USA) at 37°C in a humidified atmosphere of 5% CO_2_ in air in Dulbecco’s modified Eagle’s medium (DMEM) supplemented with 20% (v/v) fetal bovine serum, 1% (v/v) nonessential amino acid solution, 100 unit.mL^−1^ penicillin and 100 µg.mL^−1^ streptomycin. The Caco-2 cells were cultured for one week, and the experiment was conducted after their differentiation. The cells were exposed to the ER stress inducers brefeldin A (0.3 µg/mL) (Sigma), and tunicamycin (0.75 µg/mL) (Sigma) for 12 hours [36]. Additional groups of cells consisted in ER-stressed cells which were incubated for 12 hours in reduced-serum DMEM containing a defined amount of L-glutamine (5 mM or 10 mM) [47].

### Cell Viability Assay

The cell viability was assessed by the mitochondrial function, measured by MTT reduction activity as previously reported [48]. Briefly, cells were seeded in a 24-well plate and incubated with ER stressors (brefeldin A or tunicamycin) with or without glutamine 5 mM and 10 mM. After 12 h, the cells were incubated with 0.5 mg/mL MTT (Sigma) for 3 h at 37°C. Subsequently, the media were aspirated and the cells were lysed dimethyl sulfoxide, where after the absorbance was read at 560 nm, with background subtraction at 650 nm, using a microplate reader (Bio-Rad Laboratories, Veenendaal, The Netherlands).

### Biochemical Markers of Oxidative Stress

Oxidative stress was determined by measuring the concentration of TBARS and the GSSG/GSH ratio. The amount of aldehydic products generated by lipid peroxidation was quantified by the thiobarbituric acid reaction using 3 mg of protein per sample [49]. Results were referred as TBARS. The samples were incubated at 90°C for 30 min after adding 500 µL of 0.67% thiobarbituric acid in 10% trichloroacetic acid, them centrifuged at 2,000 g for 15 min at 4°C. Spectrophotometric absorbance was determined in the supernatant at 535 nm.

GSSG and GSH analysis was performed by the method of Hissin and Hilf [50]. Briefly, 250 mg of tissue was homogenised in 0.1 M sodium phosphate 5 mM EDTA buffer (pH 8.0) with 25% phosphoric acid at a proportion of 1∶20. The mixture was centrifuged at 100,000 g for 30 min at 4°C, the supernatant was collected and 500 µL were diluted with 4.5 mL of buffer. For GSH assay, to 100 µL supernatant 1.8 mL phosphate-EDTA buffer and 100 µL O-phthalaldehyde were added. After incubating for 15 min at 4°C, a spectrofluorometric reading was obtained at an excitation wavelength of 350 nm and an emission wavelength of 420 nm. For GSSG assay, 500 µL of the sample supernatant was incubated with 200 µL of 0.04 M N-ethylmaleimide for 30 min; to this mixture 4.5 mL of 0.1 N NaOH was added. A 100 µL portion of this mixture was then processed using the procedure outlined above for GSH assay.

### Caspase Activities

Lysates were prepared by homogenizing colon tissue in 0.25 mM sucrose, 1 mM EDTA, 10 mM Tris and a protease inhibitor cocktail (Roche Diagnostics GmbH, Mannheim, Germany) [51]. The lysates were then centrifuged at 14,000 g for 10 min at 4°C, and supernatants (50 µg protein) were incubated for 1 h at 37°C in 4-(2-hydroxyethyl)-1-piperazineethanesulfonic acid (HEPES) buffer containing 100 µM concentrations of the specific fluorogenic substrates 7-amino-4-methylcoumarin N-acetyl-L-aspartyl-Lglutamyl-L-valyl-l-aspartic acid amide (Ac-DEVD-AMC), 7-amino-4-methylcoumarin N-acetyl-l-leucyl-l-glutamyl-l-histidyl-l-aspartic acid amide (Ac-LEHD-AMC), and N-acetyl-Ile-Glu-Thr-Asp-7-amino-4-trifluoromethylcoumarin (Ac-IEDT-AFC) for caspase-3, caspase-9 and caspase-8, respectively) [52], [53]. Cleavage of the caspase substrates was monitored using a spectrofluorimeter (Hitachi F-2000 fluorimeter, Hitachi LTD, Tokyo, Japan) at excitation/emission wavelengths of 360/460 nm for caspase-9, 400/505 nm for caspase-8, and 380/460 nm for caspase-3, respectively. Activity was expressed as fluorescence units per milligram of protein per min of incubation.

### Western Blot Analysis

Western blot analyses were performed on cytosolic and nuclear extracts of colon tissue and Caco-2 cells. Nuclear extracts were prepared from colon homogenates as described previously [2], [21]. Briefly, 100 mg of colon from all animals were homogenized in 5×10^−4^ L of buffer A (0.01 M Hepes- KOH pH 7.9, 250 g/L glycerol, 0.420 M NaCl, 0.0015 M MgCl_2_, 2×10^−4^ M EDTA, 5×10^−4^ M DTT, 2×10^−4^ M PMSF) and a phosphatase inhibitor cocktail (Roche) to disrupt extracellular matrix and cellular membranes. Homogenates were centrifuged at 1,000 g for 10 min at 4°C. The pellet was resuspended in 2.5×10^−4^ L of buffer B (0.02 M NaCl Hepes- KOH pH 7.9, 250 g/L glycerol, 0.42 M NaCl, 15×10^−4^ M MgCl_2_, 2×10^−4^ M EDTA, 5×10^−4^ M DTT, 2×10^−4^ M PMSF) homogenized and incubated at 4°C for 30 min. Cellular debris was removed by centrifugation at 14,000 g for 15 min at 4°C. The supernatant fraction containing DNA binding proteins was recollected and stored at −80°C in aliquots until use. Cytosolic extracts were prepared by colon tissue homogenization in 0.25 mM sucrose, 1 mM EDTA, 10 mM Tris and a phosphatase and 1% protease inhibitor cocktail (Roche) [2]. The homogenate was centrifuged at 4°C for 30 min at 13,000 g. The supernatant fraction was recollected and stored at −80°C in aliquots until use. Caco-2 cells seeded on 60 mm plastic dishes were treated with glutamine and/or an ER stress inducers for a certain period of time, and total protein was recovered after washing with PBS (Sigma). The recovered sample was centrifuged at 12,000 g for 10 min at 4°C, and the resulting pellet was dissolved by RIPA buffer (20 mM Tris-HCl (pH 7.6), 2 mM EDTA, 150 mM NaCl, 1% Triton X-100, 1% Na-deoxycholate, 0.1% SDS, 1.0 mM DTT) with protease inhibitors (1 mM benzamidine-HCl, 1 mM PMSF, and 5 µg/mL each pepstatin, aprotinin, and leupeptin), and phosphatase inhibitors (5 mM sodium fluoride, 5 mM sodium phosphate, 10 mM sodium pyrophosphate, 10 mM sodium molybdate, 5 mM EDTA, and 5 mM EGTA). Protein concentration was measured by the Bradford assay. Equal amounts of protein (10–50 µg) were separated by 9–12% sodium dodecyl sulphate (SDS)-polyacrylamide gel electrophoresis for 1.5 h at 100 V and then blotted on polyvinylidene fluoride (PVDF) membranes (Amersham Pharmacia, Little Chalfont, UK). The membranes were then blocked with 5% non-fat dry milk in phosphate buffered saline buffer containing 0.05% Tween 20 (PBST) for 1 hour at room temperature and probed overnight at 4°C with polyclonal anti-Bax, Bcl-2, Bcl-xL, poly(ADP-ribose) polymerase-1 (PARP-1), cytochrome c, transcription factor CHOP/GADD153 (Santa Cruz Biotechnology, Santa Cruz, CA, USA), JNK, phospho-JNK, BiP/glucose-regulated protein 78 (GRP78), p53, phospho-p53, cleaved caspase-3 (Cell Signaling Technology, Danvers, MA, USA), calpain-1, caspase-12 and phospho-IRE1 (Abcam, Cambridge, UK) antibodies at 1∶200–1∶1,000 dilution with PBST containing 3% non-fat dry milk. Equal loading of protein was demonstrated by probing the membranes with a rabbit anti lamin-B polyclonal antibody (1∶200 Santa Cruz Biotechnology) or rabbit anti-β-actin polyclonal antibody (1∶1,000 Sigma). After washing with PBST, the membranes were incubated for 1 h at room temperature with secondary HRP conjugated antibody (Dako, Glostrup, Denmark, 1∶4,000), and visualized using ECL detection kit (Amersham Pharmacia, Uppsala, Sweden). The density of the specific bands was quantified with an imaging densitometer (Scion Image, Maryland, MA, USA).

### Real-time Quantitative RT-PCR

Total RNA was extracted and reverse transcribed using High- Capacity cDNA Archive Kit (Applied Biosystems, Foster City, CA). cDNA was amplified using TaqMan Universal PCR Master Mix (Applied Biosystems) on a Step One Plus (Applied Biosystems). TaqMan primers and probes for ATF6 (GenBank accession no BC168890.1 and Rn01490844_m1), ATF4 (GenBank accession no AF252627.1 and Rn00824644_g1), CHOP (GenBank accession no AW916370.1 and Rn00492098_g1), BiP (GenBank accession no M14050.1 and Rn00565250_m1), and glyceraldehyde-3-phosphate dehydrogenase (GenBank accession no X02231.1 and Rn99999916_s1) genes were derived from the commercially available TaqMan Gene Expression Assays (Applied Biosystems). Spliced XBP-1 mRNA was also determined by real-time PCR analysis using the following set of primers and Power SYBR Green PCR Master Mix (Applied Biosystems): CTGAGTCCGAATCAGGTGCAG (original CAG sequence was mutated to AAT to reduce the background signal from unspliced XBP-1) and ATCCATGGGGAAGATGTTCTGG [54]. Relative changes in expression levels were determined using the 2^−ΔΔCT^ method [55]. The cycle number at which the transcripts were detectable (CT) was normalized to the cycle number of GAPDH gene detection, referred to as ΔCT.

### Immunohistochemistry

Colonic samples were recovered; fixed in 10% buffered formalin, and embedded in paraffin. Sections (4 µm) were dewaxed and hydrated through graded ethanols, cooked in 25 mM citrate buffer, pH 6.0, in a pressure cooker for 10 min, transferred into boiling deionized water and let to cool for 20 min. Tissue sections were then treated with 3% hydrogen peroxide to inactivate endogenous peroxidase activity. The slides were incubated with rabbit polyclonal antibody BiP (Abcam) and cleaved caspase-3 (Cell Signaling) overnight at 4°C, followed by incubation with biotinylated second antibody (Biotinylated Anti-Rabbit IgG; Vector Laboratories, Burlingame, CA) for 1 hour at room temperature. After 45 min of avidin-biotin amplification (ABC Standard; Vector Laboratories), samples were incubated with the substrate 0.1% 3′,3′-diaminobenzidine (DAB/Ni Substrate; Vector Laboratories) at room temperature for 10 min. The nuclei were lightly counter stained with hematoxylin solution [53]. Pathological findings were assessed by one of the authors blinded to the group allocations.

### Double Immunofluorescence

For immunofluorescent double staining, serial colonic sections were dewaxed in xylene and rehydrated in graded ethanol to distilled water, do not allowing slides to dry at any time during this process. Heat-mediated antigen was performed in a cooker filled with 1 mM EDTA (pH = 8.0). Sections were brought to a boil and then maintain at a sub-boiling temperature for 15 min. All subsequent incubations with immunochemicals were performed in a humidified chamber.

After unmasking and after blocking the nonspecific binding, the sections were co-incubated with the CHOP antibody (Santa Cruz Biotechnology) and cleaved caspase-3 antibody (Cell Signaling Technology) at (1∶50 and 1∶400, respectively) dilution overnight at 4°C. After incubation with primary antibodies, samples were washed twice in PBS for 10 min at room temperature. Thereafter, the secondary antibodies donkey anti-rrabit conjugated with FITC (Jackson ImmunoResearch, Baltimore, PA) or donkey anti-mouse conjugated with Texas Red (Jackson ImmunoResearch) were applied for 2 h at 4°C. After washing in PBS, the coverslips were mounted on DakoCytomation Fluorescent Mountaing Medium (DAKO). In sections from each experimental group, the primary antibody was replaced by antibody diluent to assess for nonspecific binding of the secondary antibody [56]. The preparations were analyzed with an inverted fluorescent microscope (Nikon Eclipse Ti).

### Statistical Analysis

Data were analyzed using an analysis of variance (ANOVA) with repeated measures for time, colitis and treatment with glutamine. Bonferroni post hoc analysis was used where appropriate. P<0.05 was considered statistically significant. SPSS+ version 14.0 statistical software was used.

## References

[1] Fillmann H, Kretzmann NA, San-Miguel B, Llesuy S, Marroni N, et al (2007) Glutamine inhibits over-expression of pro-inflammatory genes and down-regulates the nuclear factor kappaB pathway in an experimental model of colitis in the rat. Toxicology 236: 217–226. 17543437 10.1016/j.tox.2007.04.012

[2] Kretzmann NA, Fillmann H, Mauriz JL, Marroni CA, Marroni N, et al (2008) Effects of glutamine on proinflammatory gene expression and activation of nuclear factor kappa B and signal transducers and activators of transcription in TNBS-induced colitis. Inflamm Bowel Dis 14: 1504–1513. 18623154 10.1002/ibd.20543

[3] Kaser A, Martínez-Naves E, Blumberg RS (2010) Endoplasmic reticulum stress: implications for inflammatory bowel disease pathogenesis. Curr Opin Gastroenterol 26: 318–326. 20495455 10.1097/MOG.0b013e32833a9ff1 PMC4592137

[4] Kaser A, Blumberg RS (2011) Autophagy, microbial sensing, endoplasmic reticulum stress, and epithelial function in inflammatory bowel disease. Gastroenterology 140: 1738–1747. 21530740 10.1053/j.gastro.2011.02.048 PMC4592160

[5] Ron D, Walter P (2007) Signal integration in the endoplasmic reticulum unfolded protein response. Nat Rev Mol Cell Biol 8: 519–529. 17565364 10.1038/nrm2199

[6] Zhang K, Kaufman RF (2008) From endoplasmic-reticulum stress to the inflammatory response. Nature 454: 455–462. 18650916 10.1038/nature07203 PMC2727659

[7] Malhi H, Kaufman RJ (2011) Endoplasmic reticulum stress in liver disease. J Hepatol 54: 795–809. 21145844 10.1016/j.jhep.2010.11.005 PMC3375108

[8] Gardner BM, Walter P (2011) Unfolded proteins are Ire1-activating ligands that directly induce the unfolded protein response. Science 30: 1891–1894. 10.1126/science.1209126 PMC3202989 21852455

[9] Kaser A, Lee AH, Franke A, Glickman JN, Zeizzing S, et al (2008) XBP1 links ER stress to intestinal inflammation and confers genetic risk for human inflammatory bowel disease. Cell 134: 743–756. 18775308 10.1016/j.cell.2008.07.021 PMC2586148

[10] Bogaert S, de Vos M, Olievier K, Peeters H, Elewaut B, et al. (2011) Involvement of endoplasmic reticulum in inflammatory bowel disease: a different implication for colonic and ileal disease?. PLoS ONE 6, e25589. 10.1371/journal.pone.0025589 PMC3196494 22028783

[11] Kaser A, Blumberg RS (2009) Endoplasmic reticulum stress in the intestinal epithelium and inflammatory bowel disease. Semin Immunol 21: 156–163. 19237300 10.1016/j.smim.2009.01.001 PMC4736746

[12] Zingarelli B, Hake PW, Burroughs TJ, Piraino G, O’connor M, et al (2004) Activator protein-1 signalling pathway and apoptosis are modulated by poly(ADP- ribose) polymerase-1 in experimental colitis. Immunology 113: 509–517. 15554929 10.1111/j.1365-2567.2004.01991.x PMC1782595

[13] Cheung HH, Lynn Kelly N, Liston P, Korneluk RG (2006) Involvement of caspase-2 and caspase-9 in endoplasmic reticulum stress-induced apoptosis: a role for the IAPs. Exp Cell Res 312: 2347–2357. 16701639 10.1016/j.yexcr.2006.03.027

[14] Shiraishi H, Okamoto H, Yoshimura A, Yoshida H (2006) ER stress-induced apoptosis and caspase-12 activation occurs downstream of mitochondrial apoptosis involving Apaf-1. J Cell Sci 119: 3958–3966. 16954146 10.1242/jcs.03160

[15] Giriş M, Erbil Y, Oztezcan S, Olgaç V, Barbaros U, et al (2006) The effect of heme oxygenase-1 induction by glutamine on radiation-induced intestinal damage: the effect of heme oxygenase-1 on radiation enteritis. Am J Surg 191: 503–509. 16531144 10.1016/j.amjsurg.2005.11.004

[16] Kim CJ, Kovacs-Nolan JA, Yang C, Archbold T, Fan MZ, et al (2010) L-Tryptophan exhibits therapeutic function in a porcine model of dextran sodium sulfate (DSS)-induced colitis. J Nutr Biochem 21: 468–475. 19428234 10.1016/j.jnutbio.2009.01.019

[17] Wang WW, Qiao SY, Li DF (2008) Amino acids and gut function. Amino Acids 37: 105–110. 18670730 10.1007/s00726-008-0152-4

[18] Mondello S, Galuppo M, Mazzon E, Domenico I, Mondello P, et al (2010) Glutamine treatment attenuates the development of ischaemia/reperfusion injury of the gut. Eur J Pharmacol 643: 304–315. 20599905 10.1016/j.ejphar.2010.06.044

[19] Xue H, Sufit AJ, Wischmeyer PE (2011) Glutamine therapy improves outcome of *in vitro* and *in vivo* experimental colitis models. JPEN J Parenter Enteral Nutr 35: 188–197. 21378248 10.1177/0148607110381407

[20] Melis GC, ter Wengel N, Boelens PG, van Leeuwen PA (2004) Glutamine: recent developments in research on the clinical significance of glutamine. Curr Opin Clin Nutr Metab Care 7: 59–70. 15090905 10.1097/00075197-200401000-00011

[21] Carneiro BA, Fujii J, Brito GA, Alcantara C, Oriá RB, et al (2006) Caspase and Bid involvement in *Clostridium difficile* toxin A-induced apoptosis and modulation of toxin A effects by glutamine and alanyl-glutamine *in vivo* and *in vitro* . Infect Immun 74: 81–87. 16368960 10.1128/IAI.74.1.81-87.2006 PMC1346681

[22] Papaconstantinou HT, Hwang KO, Rajaraman S, Hellmich MR, Townsend CM Jr, et al (1998) Glutamine deprivation induces apoptosis in intestinal epithelial cells. Surgery 124: 152–159. 9706133

[23] Ko YG, Kim EY, Kim T, Park H, Park HS, et al (2001) Glutamine-dependent antiapoptotic interaction of human glutaminyl tRNA synthetase with apoptosis signal-regulating kinase 1. J Biol Chem 276: 6030–6036. 11096076 10.1074/jbc.M006189200

[24] San-Miguel B, Crespo I, Kretzmann NA, Mauriz JL, Marroni N, et al (2010) Glutamine prevents fibrosis development in rats with colitis induced by 2,4,6-trinitrobenzene sulfonic acid. J Nutr 140: 1065–1071. 20410082 10.3945/jn.110.121525

[25] Kim CJ, Kovacs-Nolan J, Yang C, Archbold T, Fan MZ, et al (2009) L-cysteine supplementation attenuates local inflammation and restores gut homeostasis in a porcine model of colitis. Biochim Biophys Acta 1790: 1161–1169. 19520150 10.1016/j.bbagen.2009.05.018

[26] Bennett HL, Fleming JT, ÓPrey J, Ryan KM, Leung HY (2010) Androgens modulate autophagy and cell death via regulation of the endoplasmic reticulum chaperone glucose-regulated protein 78/BiP in prostate câncer cells. Cell Death Dis 1: e72. 21364676 10.1038/cddis.2010.50 PMC3032338

[27] Martín AR, Villegas I, La Casa C, de la Lastra CA (2004) Resveratrol, a polyphenol found in grapes, suppresses oxidative damage and stimulates apoptosis during early colonic inflammation in rats. Biochem Pharmacol 67: 1399–1410. 15013856 10.1016/j.bcp.2003.12.024

[28] Mazzon E, Esposito E, Crisafulli C, Riccardi L, Muià C, et al (2006) Melatonin modulates signal transduction pathways and apoptosis in experimental colitis. J Pineal Res 41: 363–373. 17014694 10.1111/j.1600-079X.2006.00378.x

[29] Namba T, Tanaka K, Ito Y, Ishihara T, Hoshino T, et al (2009) Positive role of CCAAT/enhancer-binding protein homologous protein, a transcription factor involved in the endoplasmic reticulum stress response in the development of colitis. Am J Pathol 174: 1786–1798. 19359519 10.2353/ajpath.2009.080864 PMC2671267

[30] Nielsen OH, Berild D, Ahnfelt-Rønne I (1994) *In vitro* superoxide production by peripheral neutrophils from patients with inflammatory bowel disease. Mediators Inflamm 3: 161–164. 18472936 10.1155/S0962935194000219 PMC2367029

[31] Erbil Y, Giriş M, Abbasoğlu SD, Barbaros U, Yanik BT, et al (2007) Effect of heme oxygenase-1 induction by octreotide on TNBS-induced colitis. J Gastroenterol Hepatol 22: 1852–1858. 17914959 10.1111/j.1440-1746.2007.04838.x

[32] Tavender TJ, Bulleid NJ (2010) Molecular mechanisms regulating oxidative activity of the Ero1 family in the endoplasmic reticulum. Antioxid Redox Signal 13: 1177–1189. 20486761 10.1089/ars.2010.3230

[33] Csala M, Margittai E, Bánhegyi G (2010) Redox control of endoplasmic reticulum function. Antioxid Redox Signal 13: 77–108. 20001734 10.1089/ars.2009.2529

[34] Shkoda A, Ruiz PA, Daniel H, Kim SC, Rogler G, et al (2007) Interleukin-10 blocked endoplasmic reticulum stress in intestinal epithelial cells: impact on chronic inflammation. Gastroenterology 132: 190–207. 17241871 10.1053/j.gastro.2006.10.030

[35] Zinszner H, Kuroda M, Wang X, Batchvarova N, Lightfoot RT, et al (1998) CHOP is implicated in programmed cell death in response to impaired function of the endoplasmic reticulum. Genes Dev 12: 982–995. 9531536 10.1101/gad.12.7.982 PMC316680

[36] Natsume Y, Ito S, Satsu H, Shimizu M (2009) Protective effect of quercetin on ER stress caused by calcium dynamics dysregulation in intestinal epithelial cells. Toxicology 258: 164–175. 19428936 10.1016/j.tox.2009.01.021

[37] Hayashi T, Saito A, Okuno S, Ferrand-Drake M, Chan PH (2003) Induction of GRP78 by ischemic preconditioning reduces endoplasmic reticulum stress and prevents delayed neuronal cell death. J Cereb Blood Flow Metab 23: 949–961. 12902839 10.1097/01.WCB.0000077641.41248.EA

[38] Kishi S, Shimoke K, Nakatani Y, Shimada T, Okumura N, et al (2010) Nerve growth factor attenuates 2-deoxy-D-glucose-triggered endoplasmic reticulum stress-mediated apoptosis via enhanced expression of GRP78. Neurosci Res 66: 14–21. 19766678 10.1016/j.neures.2009.09.003

[39] Nakagawa T, Yuan J (2000) Cross-talk between two cysteine protease families: activation of caspase-12 by calpain in apoptosis. J Cell Biol 150: 887–894. 10953012 10.1083/jcb.150.4.887 PMC2175271

[40] Martinez JA, Zhang Z, Svetlov SI, Hayes RL, Wang KK, et al (2010) Calpain and caspase processing of caspase-12 contribute to the ER stress-induced cell death pathway in differentiated PC12 cells. Apoptosis 15: 1480–1493. 20640600 10.1007/s10495-010-0526-4

[41] Tan Y, Dourdin N, Wu C, De Veyra T, Elce JS, et al (2006) Ubiquitous calpains promote caspase-12 and JNK activation during endoplasmic reticulum stress-induced apoptosis. J Biol Chem 281: 16016–16024. 16597616 10.1074/jbc.M601299200

[42] Tardieu D, Jaeg JP, Deloly A, Corpet DE, Cadet J, et al (2000) The COX-2 inhibitor nimesulide suppresses superoxide and 8-hydroxy-deoxyguanosine formation, and stimulates apoptosis in mucosa during early colonic inflammation in rats. Carcinogenesis 21: 973–976. 10783320 10.1093/carcin/21.5.973

[43] Yue G, Lai PS, Yin K, Nagele RG, Liu X, et al (2001) Colon epithelial cell death in 2,4,6-trinitrobenzenesulfonic acid-induced colitis is associated with increased inducible nitric-oxide synthase expression and peroxynitrite production. J Pharmacol Exp Ther 297: 915–925. 11356911

[44] Tuñón MJ, San Miguel B, Crespo I, Jorquera F, Santamaría E, et al (2011) Melatonin attenuates apoptotic liver damage in fulminant hepatic failure induced by the rabbit hemorrhagic disease virus. J Pineal Res 50: 38–45. 20964705 10.1111/j.1600-079X.2010.00807.x

[45] Morris GP, Beck PL, Herridge MS (1989) Hapten-induced model of chronic inflammation and ulceration in the rat colon. Gastroenterology 96: 795–803. 2914642

[46] Segui J, Gironella M, Sanz M, Granell S, Gil F, et al (2004) Superoxide dismutase ameliorates TNBS-induced colitis by reducing oxidative stress, adhesion molecule expression, and leukocyte recruitment into the inflamed intestine. J Leukoc Biol 76: 537–544. 15197232 10.1189/jlb.0304196

[47] Lenaerts K, Mariman E, Bouwman F, Renes J (2005) Differentiation stage-dependent preferred uptake of basolateral (systemic) glutamine into Caco-2 cells results in its accumulation in proteins with a role in cell–cell interaction. FEBS Journal 272: 3350–3364. 15978041 10.1111/j.1742-4658.2005.04750.x

[48] Crespo I, García-Mediavilla MV, Gutiérrez B, Sánchez-Campos S, Tuñón MJ, et al (2008) A comparison of the effects of quercetin and kaempferol on cytokine-induced proinflammatory status of cultured human endothelial cells. Br J Nutr 100: 968–976. 18394220 10.1017/S0007114508966083

[49] Pastor A, Collado PS, Almar M, González-Gallego J (1996) Microsomal function in biliary obstructed rats: Effects of S-adenosylmethionine. J Hepatol 24: 353–359. 8778204 10.1016/s0168-8278(96)80016-x

[50] Hissin PJ, Hilf R (1976) A fluorometric method for determination of oxidized and reduced glutathione in tissues. Anal Biochem 74: 214–226. 962076 10.1016/0003-2697(76)90326-2

[51] San-Miguel B, Alvarez M, Culebras JM, González-Gallego J, Tuñón MJ (2006) N-acetyl-cysteine protects liver from apoptotic death in an animal model of fulminant hepatic failure. Apoptosis 11: 1945–1957. 17021698 10.1007/s10495-006-0090-0

[52] Mauriz JL, González P, Jorquera F, Olcoz JL, González-Gallego J (2003) Caspase inhibition does not protect against liver damage in hemorrhagic shock. Shock 19: 33–37. 12558141 10.1097/00024382-200301000-00007

[53] Tuñón MJ, San Miguel B, Crespo I, Jorquera F, Santamaría E, et al (2011) Melatonin attenuates apoptotic liver damage in fulminant hepatic failure induced by the rabbit hemorrhagic disease virus. J Pineal Res 50: 38–45. 20964705 10.1111/j.1600-079X.2010.00807.x

[54] Lipson KL, Ghosh R, Urano F (2008) The role of IRE1α in the degradation of insulin mRNA in pancreatic β-cells. PLoS ONE 3: 1–7. 10.1371/journal.pone.0001648 PMC2241665 18286202

[55] Crespo I, García-Mediavilla MV, Almar M, González P, Tuñón MJ, et al (2008) Differential effects of dietary flavonoids on reactive oxygen and nitrogen species generation and changes in antioxidant enzyme expression induced by proinflammatory cytokines in Chang Liver cells. Food Chem Toxicol 46: 1555–1569. 18234413 10.1016/j.fct.2007.12.014

[56] García-Mediavilla MV, Pisonero-Vaquero S, Lima-Cabello E, Benedicto I, Majano PL, et al (2012) Liver X receptor α-mediated regulation of lipogenesis by core and NS5A proteins contributes to HCV-induced liver steatosis and HCV replication. Lab Invest 92: 1191–1202. 22641099 10.1038/labinvest.2012.88

