# Expression of Concern: Glutamine Treatment Attenuates Endoplasmic Reticulum Stress and Apoptosis in TNBS-Induced Colitis

**DOI:** 10.1371/journal.pone.0297611

**Published:** 2024-01-19

**Authors:** The PLOS ONE Editors

After this article [1] was published, concerns were raised about Fig 1 and Fig 5.

Specifically:

- In Fig 1F, the top central region of the “Control + G” panel appears similar to the bottom right region of the “TNBS + G 7D” panel.
- In Fig 5A, when levels are adjusted to visualize the background there appears to be a vertical discontinuity between the “Brefeldin A + G 10mM” and “Tunicamycin” lanes and the “Tunicamycin + G 5mM” and “Tunicamycin + G 10mM” lanes in the ATF6 panel.

The authors stated that the concern in Fig 1F was due to an error made in the preparation of the figures and provided the raw data underlying all images contained in this figure and that a replacement figure could be provided for the “TNBS + G 7D” panel.

Regarding the concern in Fig 5A the authors provided a raw blot described as the original underlying blot for this figure and stated that areas of the blot had been accidentally stained with a marker and that the background had been altered when trying to hide this. The *PLOS ONE* Editors were unable to verify that the band in the “Tunicamycin + G 10mM” lane in the underlying blot corresponded to the band in the published panel. Furthermore, the authors stated that individual quantification data for these blots was not available and was not provided for editorial review. The available underlying data for this article is available in S1 File. The *PLOS ONE* Editors remain concerned about this figure.

In light of the above concerns, the *PLOS ONE* Editors issue this Expression of Concern.

**Fig 1:**
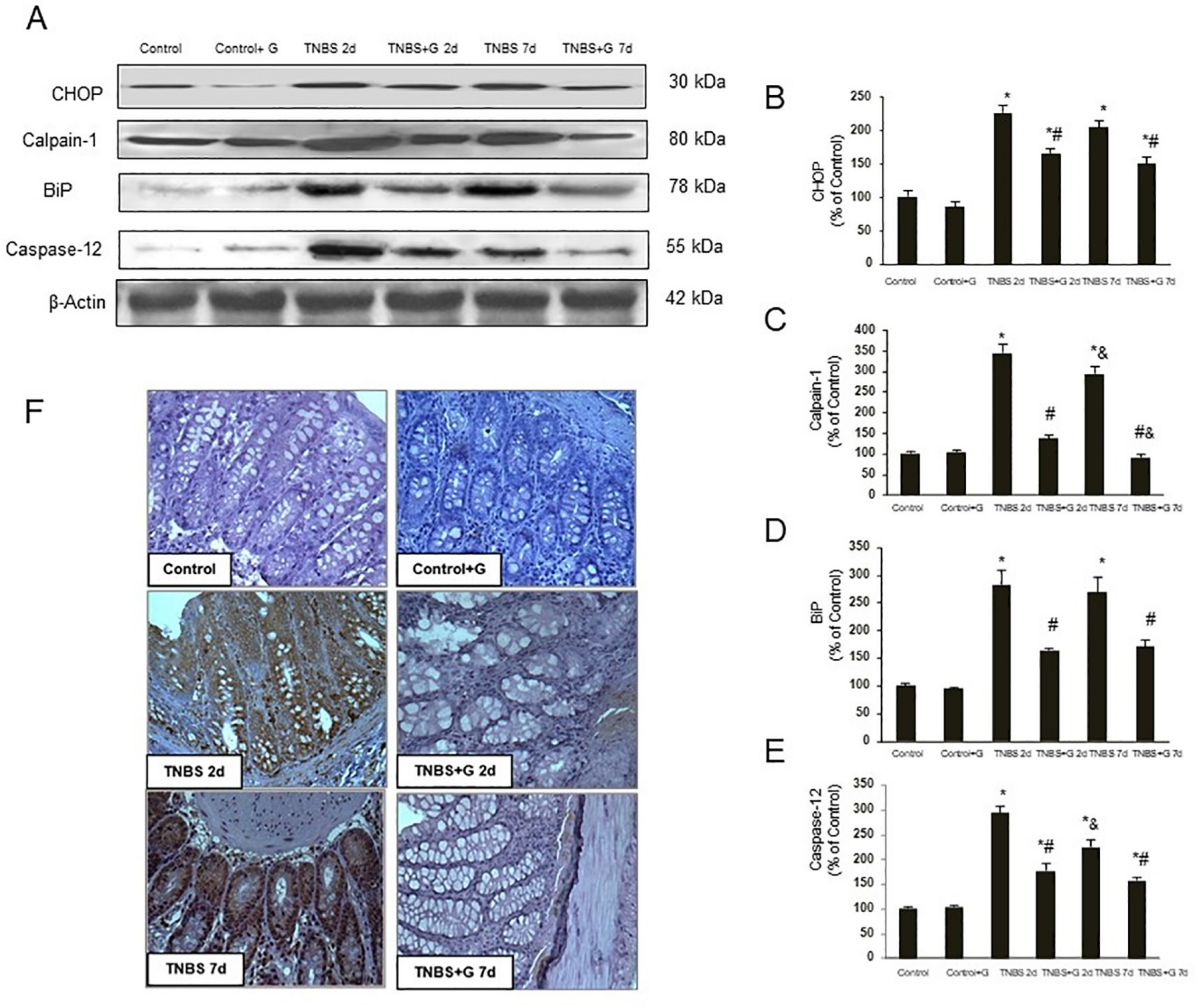
Glutamine reduces the ER stress induced by TNBS-colitis. (A–E). Protein from colonic extracts was separated by sodium dodecyl sulfate-polyacrylamide gel electrophoresis, followed by immunoblotting for CHOP, BiP, calpain-1 and caspase-12. CHOP, BiP, calpain-1 and caspase-12 were markedly expressed in rats treated with TNBS alone. However, glutamine administration partially abolished CHOP, BiP, calpain-1 and caspase-12 expression induced by TNBS. Results are representative of four independent experiments. Equal loading of proteins is illustrated by β-actin bands. (A) Representative Western-blot photographs for CHOP, calpain-1, BiP, caspase-12, and β-actin. (B) Densitometric quantification of CHOP. (C) Densitometric quantification of calpain-1. (D) Densitometric quantification of BiP. (E) Densitometric quantification of caspase-12. Data are expressed as mean ± S.E.M. from 8 rats. *P<0.05 compared with control group. ^#^P<0.05 compared with TNBS group. ^&^P<0.05 compared with same group 2 d. (F) Photomicrographs of immunohistochemistry for BiP in sections of colonic samples. Paraffin-embedded sections were immunostained with a BiP antibody. Original magnification: 200X.

## Supporting information

S1 File Available Underlying Data. (PDF)
