# Supplementary material for: Expression of Concern: Glutamine Treatment Attenuates Endoplasmic Reticulum Stress and Apoptosis in TNBS-Induced Colitis
Source: PLoS One. 2024 Jan 19;19(1):e0297611. doi: 10.1371/journal.pone.0297611 (PMC10798430; doi:10.1371/journal.pone.0297611)

**Data recovered**

**Figure 1A**

CHOP Raw material

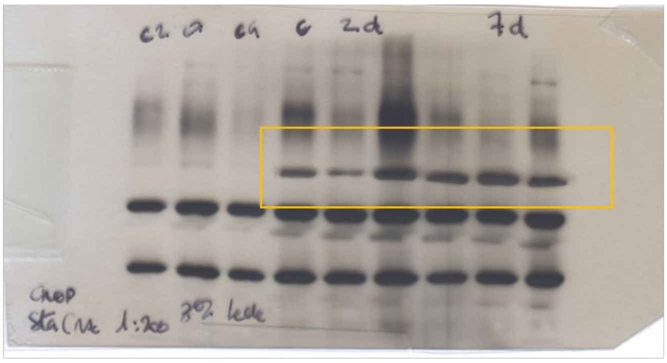


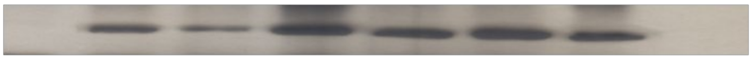

Published

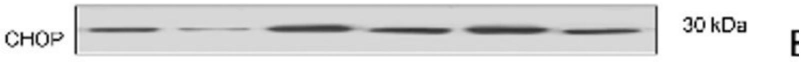


Calpain-1 Raw material

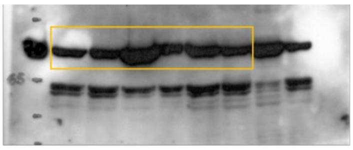

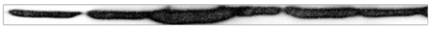

Published

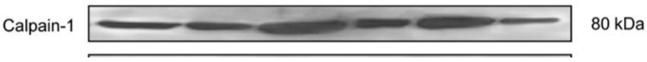

Bip Raw material

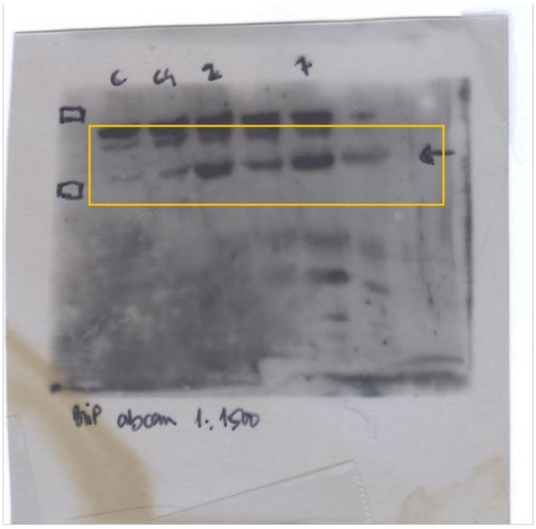


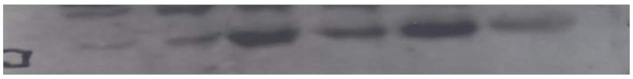

Published

BiP 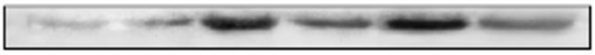 78 kDa

Caspase-12 Raw material

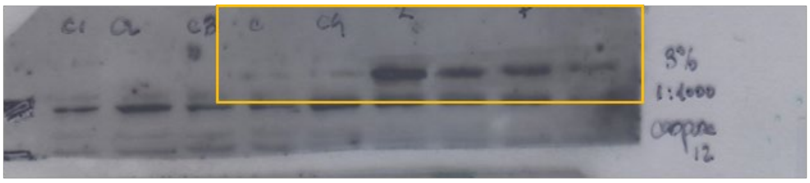


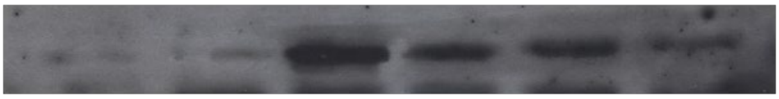

Published

Caspase-12 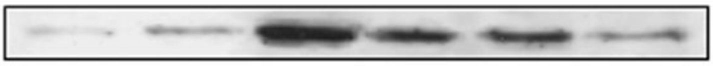 55 kDa

**Figure 1F**

Control

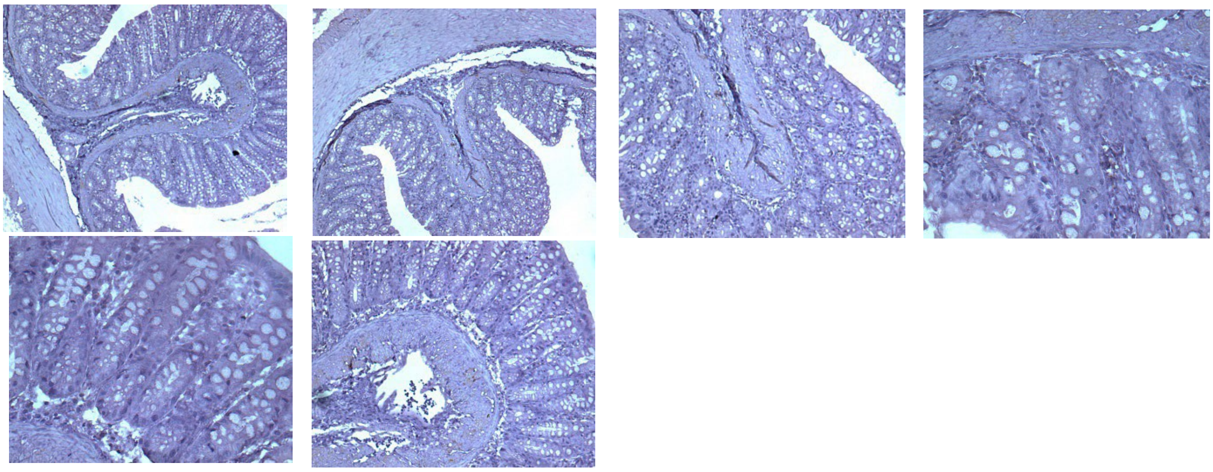

Control+G

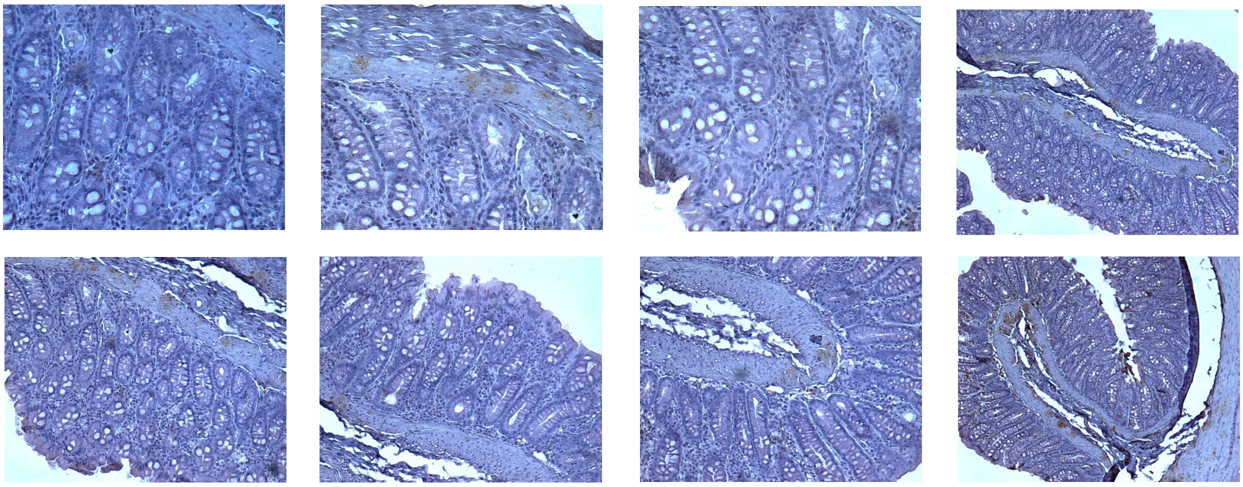

TNBS2d

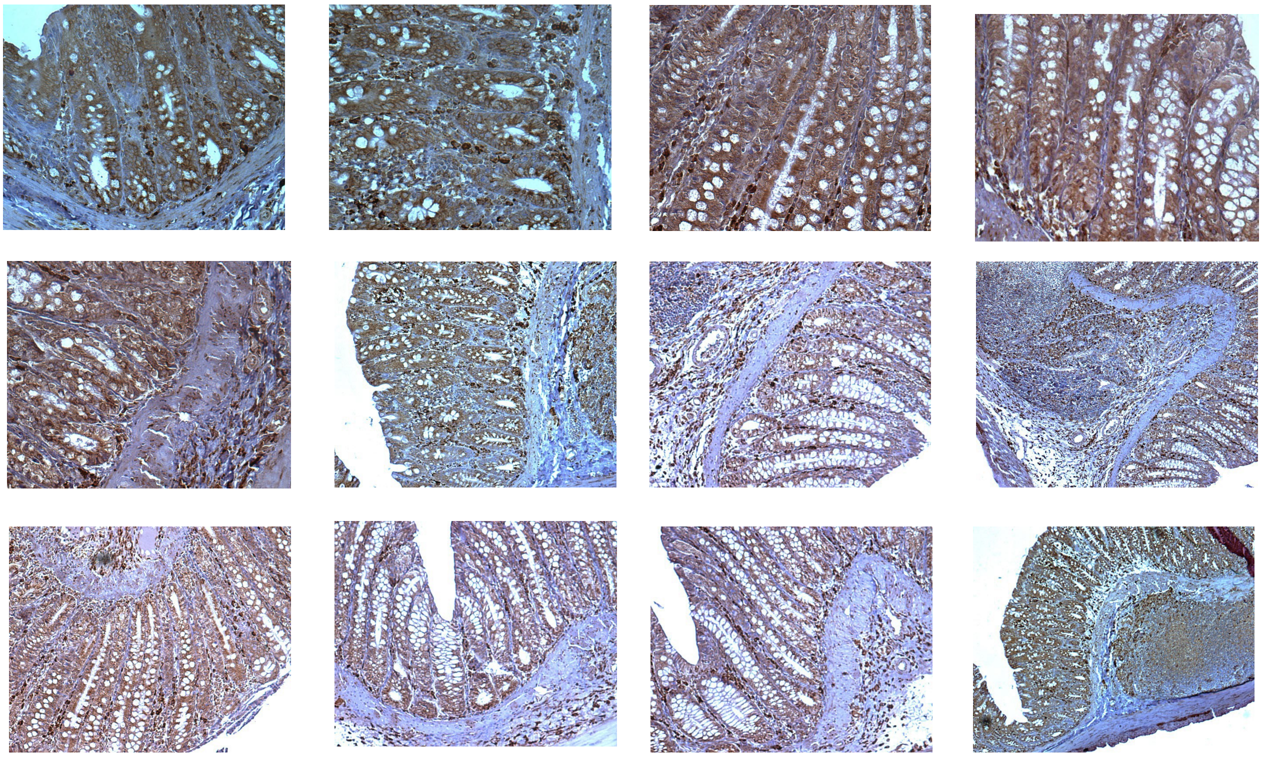

TNBS+G 2d

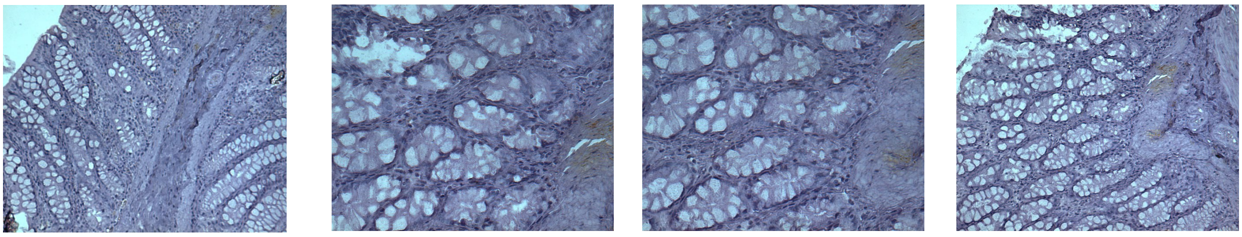

TNBS 7d

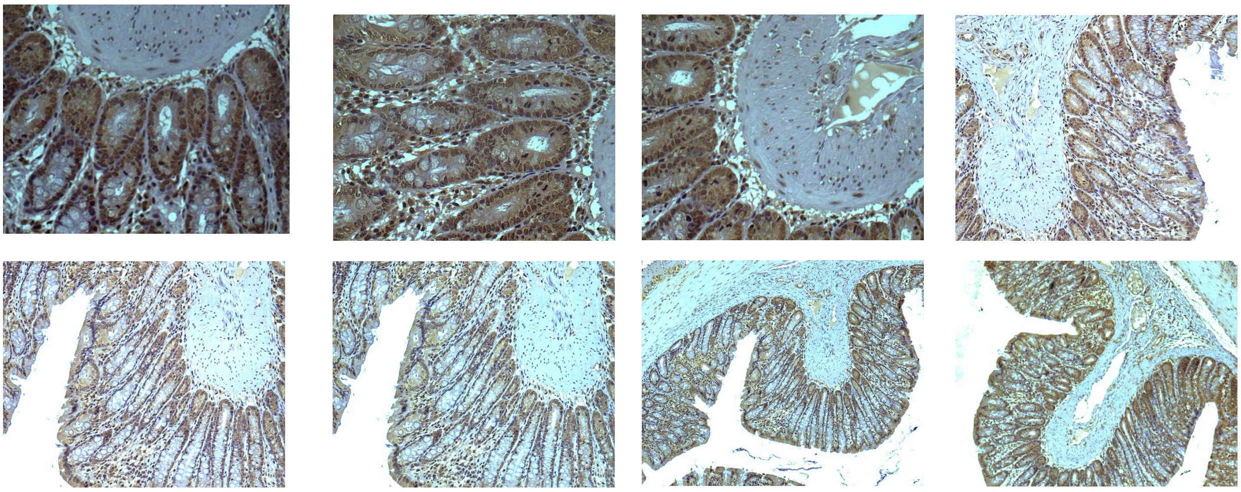

TNBS+G 7d

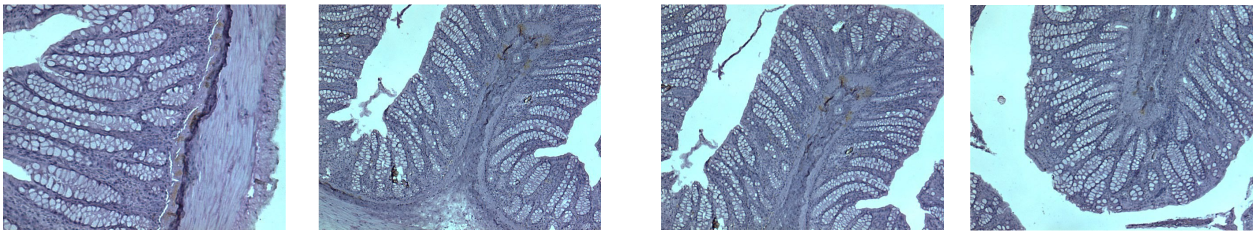

**Figure 3A**

F13 fx 1899

| | 1 | 2 | 3 | 4 | 5 | 6 | 7 | 8 | 9 | 10 | 11 | 12 | |
|---|---|---|---|---|---|---|---|---|---|---|---|---|---|
| A | 1457 | 1328 | 1389 | 1269 | 1780 | 1715 | | | | | | | 360/40,528/20 Read#1 |
| B | 1894 | 1789 | 1688 | 1964 | 1356 | 1371 | | | | | | | 360/40,528/20 Read#1 |
| C | 1433 | 1313 | 1294 | 1276 | 1274 | 1256 | | | | | | | 360/40,528/20 Read#1 |
| D | 2507 | 2647 | 1473 | 1439 | | | | | | | | | 360/40,528/20 Read#1 |
| E | 1354 | 1256 | 1311 | 1365 | | | | | | | | | 360/40,528/20 Read#1 |
| F | 1485 | 1432 | 1400 | 1346 | | | | | | | | | 360/40,528/20 Read#1 |
| G | 1276 | 1229 | 1262 | 1267 | | | | | | | | | 360/40,528/20 Read#1 |
| H | 1618 | 1578 | 1592 | 671 | | | | | | | | | 360/40,528/20 Read#1 |

| | 1 | 2 | 3 | 4 | 5 | 6 | 7 | 8 | 9 | 10 | 11 | 12 | |
|---|---|---|---|---|---|---|---|---|---|---|---|---|---|
| A | 1505 | 1388 | 1450 | 1323 | 1899 | 1805 | | | | | | | 360/40,528/20 Read#2 |
| B | 1981 | 1876 | 1957 | 1743 | 1405 | 1469 | | | | | | | 360/40,528/20 Read#2 |
| C | 1486 | 1373 | 1360 | 1341 | 1598 | 1604 | | | | | | | 360/40,528/20 Read#2 |
| D | 2807 | 2918 | 1810 | 1540 | | | | | | | | | 360/40,528/20 Read#2 |
| E | 1435 | 1345 | 1529 | 1581 | | | | | | | | | 360/40,528/20 Read#2 |
| F | 1686 | 1503 | 1609 | 1658 | | | | | | | | | 360/40,528/20 Read#2 |
| G | 1346 | 1345 | 1329 | 1361 | | | | | | | | | 360/40,528/20 Read#2 |
| H | 1698 | 2042 | 1736 | 1732 | | | | | | | | | 360/40,528/20 Read#2 |

| | 1 | 2 | 3 | 4 | 5 | 6 | 7 | 8 | 9 | 10 | 11 | 12 | |
|---|---|---|---|---|---|---|---|---|---|---|---|---|---|
| A | 1548 | 1441 | 1550 | 1454 | 2261 | 1949 | | | | | | | 360/40,528/20 Read#3 |
| B | 2011 | 2011 | 1923 | 1882 | 1512 | 1523 | | | | | | | 360/40,528/20 Read#3 |
| C | 1489 | 1496 | 1483 | 1510 | 1854 | 1698 | | | | | | | 360/40,528/20 Read#3 |
| D | 3438 | 3087 | 3036 | 1739 | | | | | | | | | 360/40,528/20 Read#3 |
| E | 1677 | 1551 | 2306 | 1751 | | | | | | | | | 360/40,528/20 Read#3 |
| F | 2696 | 1755 | 2189 | 1846 | | | | | | | | | 360/40,528/20 Read#3 |
| G | 1781 | 2149 | 1580 | 1769 | | | | | | | | | 360/40,528/20 Read#3 |

| | 1 | 2 | 3 | 4 | 5 | 6 | 7 | 8 | 9 | 10 | 11 | 12 | |
|---|---|---|---|---|---|---|---|---|---|---|---|---|---|
| A | 1645 | 1500 | 1843 | 1845 | 2963 | 2277 | | | | | | | 360/40,528/20 Read#4 |
| B | 2025 | 2047 | 2316 | 1942 | 1838 | 1625 | | | | | | | 360/40,528/20 Read#4 |
| C | 1526 | 1498 | 1901 | 2001 | 1974 | 1704 | | | | | | | 360/40,528/20 Read#4 |
| D | 3445 | 3062 | 3102 | 2222 | | | | | | | | | 360/40,528/20 Read#4 |
| E | 1728 | 1589 | 2697 | 2139 | | | | | | | | | 360/40,528/20 Read#4 |
| F | 3015 | 2321 | 2888 | 2612 | | | | | | | | | 360/40,528/20 Read#4 |
| G | 2506 | 2439 | 2098 | 2523 | | | | | | | | | 360/40,528/20 Read#4 |
| H | 2432 | 3093 | 3190 | 1687 | | | | | | | | | 360/40,528/20 Read#4 |

| | 1 | 2 | 3 | 4 | 5 | 6 | 7 | 8 | 9 | 10 | 11 | 12 | |
|---|---|---|---|---|---|---|---|---|---|---|---|---|---|
| A | 1578 | 1506 | 2309 | 2550 | 3676 | 2750 | | | | | | | 360/40,528/20 Read#5 |
| B | 2179 | 2184 | 2858 | 2033 | 1984 | 1803 | | | | | | | 360/40,528/20 Read#5 |
| C | 1658 | 1610 | 2483 | 2531 | 1972 | 1874 | | | | | | | 360/40,528/20 Read#5 |
| D | 3451 | 3052 | 3098 | 3025 | | | | | | | | | 360/40,528/20 Read#5 |
| E | 1712 | 1573 | 2682 | 2644 | | | | | | | | | 360/40,528/20 Read#5 |
| F | 3010 | 3039 | 2881 | 2742 | | | | | | | | | 360/40,528/20 Read#5 |
| G | 2490 | 2428 | 2593 | 2533 | | | | | | | | | 360/40,528/20 Read#5 |
| H | 3208 | 3089 | 3193 | 1785 | | | | | | | | | 360/40,528/20 Read#5 |

| | 1 | 2 | 3 | 4 | 5 | 6 | 7 | 8 | 9 | 10 | 11 | 12 | |
|---|---|---|---|---|---|---|---|---|---|---|---|---|---|
| A | 1589 | 1587 | 2856 | 2674 | 3747 | 3459 | | | | | | | 360/40,528/20 Read#6 |
| B | 2667 | 2579 | 2884 | 2025 | 1997 | 1978 | | | | | | | 360/40,528/20 Read#6 |
| C | 1605 | 1687 | 2802 | 2809 | 2008 | 1997 | | | | | | | 360/40,528/20 Read#6 |
| D | 3469 | 3103 | 3109 | 3165 | | | | | | | | | 360/40,528/20 Read#6 |
| E | 2096 | 2053 | 2676 | 2647 | | | | | | | | | 360/40,528/20 Read#6 |
| F | 3027 | 3045 | 2865 | 2731 | | | | | | | | | 360/40,528/20 Read#6 |
| G | 2475 | 2405 | 2579 | 2515 | | | | | | | | | 360/40,528/20 Read#6 |
| H | 3258 | 3106 | 3198 | 1789 | | | | | | | | | 360/40,528/20 Read#6 |

| | 1 | 2 | 3 | 4 | 5 | 6 | 7 | 8 | 9 | 10 | 11 | 12 | |
|---|---|---|---|---|---|---|---|---|---|---|---|---|---|
| A | 1548 | 1489 | 2961 | 2663 | 3955 | 4065 | | | | | | | 360/40,528/20 Read#11 |
| B | 2647 | 2563 | 2875 | 2022 | 2356 | 2301 | | | | | | | 360/40,528/20 Read#11 |
| C | 2017 | 2087 | 2843 | 2839 | 2249 | 2243 | | | | | | | 360/40,528/20 Read#11 |
| D | 3606 | 3192 | 3204 | 3227 | | | | | | | | | 360/40,528/20 Read#11 |
| E | 2378 | 2326 | 2666 | 2622 | | | | | | | | | 360/40,528/20 Read#11 |
| F | 3097 | 3118 | 2889 | 2748 | | | | | | | | | 360/40,528/20 Read#11 |
| G | 2409 | 2327 | 2572 | 2528 | | | | | | | | | 360/40,528/20 Read#11 |
| H | 3361 | 3172 | 3307 | 1308 | | | | | | | | | 360/40,528/20 Read#11 |

| | 1 | 2 | 3 | 4 | 5 | 6 | 7 | 8 | 9 | 10 | 11 | 12 | |
|---|---|---|---|---|---|---|---|---|---|---|---|---|---|
| A | 1589 | 1548 | 2977 | 2663 | 4011 | 4126 | | | | | | | 360/40,528/20 Read#12 |
| B | 2655 | 2564 | 2877 | 2018 | 2300 | 2311 | | | | | | | 360/40,528/20 Read#12 |
| C | 2178 | 2854 | 2852 | 2843 | 2249 | 2250 | | | | | | | 360/40,528/20 Read#12 |
| D | 3620 | 3212 | 3212 | 3252 | | | | | | | | | 360/40,528/20 Read#12 |
| E | 2487 | 2430 | 2673 | 2614 | | | | | | | | | 360/40,528/20 Read#12 |
| F | 3118 | 3132 | 2887 | 2756 | | | | | | | | | 360/40,528/20 Read#12 |
| G | 3397 | 3331 | 2587 | 2524 | | | | | | | | | 360/40,528/20 Read#12 |
| H | 3370 | 3179 | 3332 | 1316 | | | | | | | | | 360/40,528/20 Read#12 |

| | 1 | 2 | 3 | 4 | 5 | 6 | 7 | 8 | 9 | 10 | 11 | 12 | |
|---|---|---|---|---|---|---|---|---|---|---|---|---|---|
| A | 1578 | 1694 | 2984 | 2667 | 4053 | 4161 | | | | | | | 360/40,528/20 Read#13 |
| B | 2656 | 2570 | 2279 | 2023 | 2464 | 2514 | | | | | | | 360/40,528/20 Read#13 |
| C | 2983 | 2765 | 2869 | 2866 | 2344 | 2344 | | | | | | | 360/40,528/20 Read#13 |
| D | 3674 | 3228 | 3245 | 3280 | | | | | | | | | 360/40,528/20 Read#13 |
| E | 2486 | 2429 | 2681 | 2613 | | | | | | | | | 360/40,528/20 Read#13 |
| F | 3132 | 3167 | 2698 | 2758 | | | | | | | | | 360/40,528/20 Read#13 |
| G | 3396 | 3322 | 2586 | 2530 | | | | | | | | | 360/40,528/20 Read#13 |
| H | 3406 | 3215 | 3363 | 3421 | | | | | | | | | 360/40,528/20 Read#13 |

| | 1 | 2 | 3 | 4 | 5 | 6 | 7 | 8 | 9 | 10 | 11 | 12 | |
|---|---|---|---|---|---|---|---|---|---|---|---|---|---|
| A | 1574 | 1598 | 2918 | 2671 | 3778 | 3852 | | | | | | | 360/40,528/20 Read#7 |
| B | 2664 | 2568 | 2880 | 2022 | 2007 | 2071 | | | | | | | 360/40,528/20 Read#7 |
| C | 1787 | 1788 | 2811 | 2808 | 2556 | 2055 | | | | | | | 360/40,528/20 Read#7 |
| D | 3487 | 3103 | 3128 | 3169 | | | | | | | | | 360/40,528/20 Read#7 |
| E | 2180 | 2149 | 2669 | 2629 | | | | | | | | | 360/40,528/20 Read#7 |
| F | 3025 | 3050 | 2866 | 2731 | | | | | | | | | 360/40,528/20 Read#7 |
| G | 2457 | 2375 | 2571 | 2515 | | | | | | | | | 360/40,528/20 Read#7 |
| H | 3276 | 3107 | 3224 | 1875 | | | | | | | | | 360/40,528/20 Read#7 |

| | 1 | 2 | 3 | 4 | 5 | 6 | 7 | 8 | 9 | 10 | 11 | 12 | |
|---|---|---|---|---|---|---|---|---|---|---|---|---|---|
| A | 1574 | 1546 | 2930 | 2672 | 3826 | 3910 | | | | | | | 360/40,528/20 Read#8 |
| B | 2656 | 2567 | 2888 | 2013 | 2107 | 2061 | | | | | | | 360/40,528/20 Read#8 |
| C | 1807 | 1907 | 2813 | 2812 | 2050 | 2552 | | | | | | | 360/40,528/20 Read#8 |
| D | 3515 | 3123 | 3136 | 3176 | | | | | | | | | 360/40,528/20 Read#8 |
| E | 2291 | 2233 | 2661 | 2621 | | | | | | | | | 360/40,528/20 Read#8 |
| F | 3048 | 3078 | 2866 | 2728 | | | | | | | | | 360/40,528/20 Read#8 |
| G | 2446 | 2360 | 2576 | 2516 | | | | | | | | | 360/40,528/20 Read#8 |
| H | 3300 | 3120 | 3236 | 1875 | | | | | | | | | 360/40,528/20 Read#8 |

| | 1 | 2 | 3 | 4 | 5 | 6 | 7 | 8 | 9 | 10 | 11 | 12 | |
|---|---|---|---|---|---|---|---|---|---|---|---|---|---|
| A | 1600 | 1608 | 2940 | 2658 | 3879 | 3961 | | | | | | | 360/40,528/20 Read#9 |
| B | 2652 | 2560 | 2869 | 2015 | 2287 | 2199 | | | | | | | 360/40,528/20 Read#9 |
| C | 1980 | 1987 | 2817 | 2820 | 2510 | 2140 | | | | | | | 360/40,528/20 Read#9 |
| D | 3534 | 3148 | 3167 | 3201 | | | | | | | | | 360/40,528/20 Read#9 |
| E | 2275 | 2232 | 2665 | 2624 | | | | | | | | | 360/40,528/20 Read#9 |
| F | 3058 | 3092 | 2878 | 2728 | | | | | | | | | 360/40,528/20 Read#9 |
| G | 2419 | 2358 | 2574 | 2514 | | | | | | | | | 360/40,528/20 Read#9 |
| H | 3313 | 3138 | 3259 | 1987 | | | | | | | | | 360/40,528/20 Read#9 |

M162

| | 1 | 2 | 3 | 4 | 5 | 6 | 7 | 8 | 9 | 10 | 11 | 12 | | 0 |
|---|---|---|---|---|---|---|---|---|---|---|---|---|---|---|
| A | 175 | 171 | 160 | 173 | 179 | 193 | 194 | 251 | 5390 | 5367 | 5715 | 5893 | 360/40,460/40 Read#1 | |
| B | 277 | 167 | 160 | 159 | 193 | 191 | 196 | 190 | 5157 | 4946 | 5017 | 5539 | 360/40,460/40 Read#1 | |
| C | 178 | 168 | 174 | 170 | 296 | 178 | 210 | 193 | 5335 | 5077 | 5302 | 5608 | 360/40,460/40 Read#1 | |
| D | 178 | 165 | 519 | 171 | 183 | 178 | 313 | 191 | 5310 | 5484 | 5368 | 5380 | 360/40,460/40 Read#1 | |
| E | 172 | 168 | 173 | 171 | 178 | 176 | 225 | 206 | 5731 | 5499 | 5735 | 6058 | 360/40,460/40 Read#1 | |
| F | 170 | 164 | 188 | 172 | 174 | 207 | 5380 | 5166 | 5461 | 5414 | 5033 | 5561 | 360/40,460/40 Read#1 | |
| G | 164 | 166 | 199 | 172 | 184 | 177 | 5689 | 5638 | 5362 | 4957 | 5136 | 5657 | 360/40,460/40 Read#1 | |
| H | 173 | 165 | 174 | 174 | 184 | 183 | 6383 | 6335 | 5838 | 5861 | 4979 | 5354 | 360/40,460/40 Read#1 | |

| | 1 | 2 | 3 | 4 | 5 | 6 | 7 | 8 | 9 | 10 | 11 | 12 | | 5 |
|---|---|---|---|---|---|---|---|---|---|---|---|---|---|---|
| A | 172 | 169 | 159 | 173 | 177 | 188 | 191 | 238 | 6001 | 6087 | 6686 | 6990 | 360/40,460/40 Read#2 | |
| B | 271 | 190 | 159 | 158 | 190 | 185 | 193 | 187 | 5283 | 5007 | 4897 | 5577 | 360/40,460/40 Read#2 | |
| C | 175 | 164 | 170 | 166 | 298 | 176 | 205 | 193 | 5572 | 5588 | 6201 | 6722 | 360/40,460/40 Read#2 | |
| D | 175 | 163 | 505 | 169 | 177 | 175 | 305 | 187 | 5580 | 5702 | 5457 | 5489 | 360/40,460/40 Read#2 | |
| E | 170 | 164 | 170 | 170 | 176 | 172 | 221 | 202 | 6439 | 6244 | 6761 | 7149 | 360/40,460/40 Read#2 | |
| F | 167 | 159 | 185 | 168 | 171 | 204 | 5533 | 5286 | 5953 | 5937 | 5441 | 6052 | 360/40,460/40 Read#2 | |
| G | 160 | 162 | 193 | 167 | 179 | 175 | 5964 | 5959 | 5654 | 5196 | 5249 | 5823 | 360/40,460/40 Read#2 | |
| H | 176 | 162 | 171 | 172 | 179 | 180 | 7914 | 7430 | 6130 | 6172 | 4871 | 5231 | 360/40,460/40 Read#2 | |

| | 1 | 2 | 3 | 4 | 5 | 6 | 7 | 8 | 9 | 10 | 11 | 12 | | 10 |
|---|---|---|---|---|---|---|---|---|---|---|---|---|---|---|
| A | 172 | 168 | 157 | 170 | 176 | 187 | 189 | 233 | 6875 | 6900 | 7996 | 8460 | 360/40,460/40 Read#3 | |
| B | 265 | 220 | 155 | 158 | 188 | 185 | 189 | 185 | 5525 | 5192 | 4905 | 5549 | 360/40,460/40 Read#3 | |
| C | 174 | 164 | 169 | 163 | 295 | 173 | 204 | 189 | 6091 | 6273 | 7506 | 8089 | 360/40,460/40 Read#3 | |
| D | 174 | 161 | 197 | 167 | 176 | 174 | 304 | 184 | 5931 | 6044 | 5624 | 5706 | 360/40,460/40 Read#3 | |
| E | 167 | 163 | 166 | 167 | 174 | 171 | 209 | 201 | 7541 | 7218 | 7941 | 8518 | 360/40,460/40 Read#3 | |
| F | 164 | 159 | 183 | 167 | 169 | 201 | 5738 | 5446 | 6633 | 6661 | 6008 | 6710 | 360/40,460/40 Read#3 | |
| G | 159 | 160 | 191 | 166 | 178 | 174 | 6562 | 6526 | 5958 | 5561 | 5370 | 6116 | 360/40,460/40 Read#3 | |
| H | 175 | 161 | 170 | 169 | 178 | 177 | 9686 | 8950 | 6750 | 6848 | 4806 | 5158 | 360/40,460/40 Read#3 | |

| tiempo 60 | | | promedio |
|---|---|---|---|
| Bl | 1578 | 1694 | 1636 |
| 2,2 | 2656 | 2570 | 2613 |
| 2,3 | 2983 | 2765 | 2874 |
| 2,5 | 3674 | 3228 | 3451 |
| 2,7 | 2486 | 2429 | 2457,5 |
| 2,1 | 3132 | 3167 | 3149,5 |
| 2,11 | 3396 | 3322 | 3359 |
| 2,12 | 3406 | 3215 | 3310,5 |
| 2,19 | 2984 | 2667 | 2825,5 |
| 2,2 | 2279 | 2023 | 2151 |
| 7,2 | 2869 | 2866 | 2867,5 |
| 7,3 | 3245 | 3280 | 3262,5 |
| 7,5 | 2681 | 2613 | 2647 |
| 7,7 | 2698 | 2758 | 2728 |
| 7,1 | 2586 | 2530 | 2558 |
| 7,11 | 3363 | 3421 | 3392 |
| 7,12 | 4053 | 4161 | 4107 |
| 7,14 | 2464 | 2514 | 2489 |
| 7,16 | 2344 | 2344 | 2344 |

| | | |
|---|---|---|
| 2,2 | 684,5 | 1064,5 |
| 2,3 | 1444,5 | |
| 2,5 | 588,5 | 828 |
| 2,7 | 1067,5 | |
| 2,1 | 1555 | 1669,66667 |
| 2,11 | 2013,5 | |
| 2,12 | 1440,5 | |
| 2,19 | 1439 | 663 |
| 2,2 | 698 | |
| 7,2 | 628 | 807,5 |
| 7,3 | 987 | |
| 7,5 | 1092 | 1093,25 |
| 7,7 | 1094,5 | |
| 7,1 | 1213 | 1375,33333 |
| 7,11 | 1658 | |
| 7,12 | 1255 | |
| 7,14 | 1052 | 897,5 |
| 7,16 | 743 | |

| tiempo 5 | | promedio |
|---|---|---|
| 1505 | 1388 | 1446,5 |
| 1981 | 1876 | 1928,5 |
| 1486 | 1373 | 1429,5 |
| 2807 | 2918 | 2862,5 |
| 1435 | 1345 | 1390 |
| 1686 | 1503 | 1594,5 |
| 1346 | 1345 | 1345,5 |
| 1698 | 2042 | 1870 |
| 1450 | 1323 | 1386,5 |
| 1957 | 1743 | 1850 |
| 1360 | 1341 | 1350,5 |
| 1810 | 1540 | 1675 |
| 1529 | 1581 | 1555 |
| 1609 | 1658 | 1633,5 |
| 1329 | 1361 | 1345 |
| 1736 | 1732 | 1734 |
| 1899 | 1805 | 1852 |
| 1405 | 1469 | 1437 |
| 1598 | 1604 | 1601 |

| | C2 | C+G2 | COLITIS | COLITIS+GLUT | C7 | C+G7 | COL 7 | COL+G 7 |
|---|---|---|---|---|---|---|---|---|
| UAF | 1064,5 | 828,0 | 1669,7 | 663,0 | 807,5 | 1093,3 | 1375,3 | 897,5 |
| UAF/PROT/MIN | 774,182 | 602,182 | 1214,303 | 482,182 | 587,273 | 795,091 | 1000,242 | 652,727 |

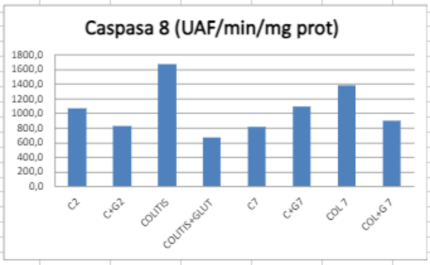

| | 1 | 2 | 3 | 4 | 5 | 6 | 7 | 8 | 9 | 10 | 11 | 12 | | 15 |
|---|---|---|---|---|---|---|---|---|---|---|---|---|---|---|
| A | 170 | 166 | 156 | 168 | 175 | 186 | 188 | 229 | 7907 | 8046 | 9509 | 10160 | 360/40,460/40 Read#4 | |
| B | 330 | 159 | 154 | 153 | 188 | 183 | 189 | 184 | 5745 | 5413 | 4950 | 5540 | 360/40,460/40 Read#4 | |
| C | 170 | 161 | 169 | 164 | 293 | 172 | 203 | 187 | 6751 | 7035 | 9062 | 9738 | 360/40,460/40 Read#4 | |
| D | 173 | 159 | 192 | 166 | 174 | 172 | 301 | 185 | 6429 | 6492 | 5815 | 6007 | 360/40,460/40 Read#4 | |
| E | 166 | 161 | 166 | 166 | 173 | 171 | 209 | 199 | 8773 | 8525 | 9401 | 10142 | 360/40,460/40 Read#4 | |
| F | 164 | 158 | 181 | 165 | 168 | 200 | 6018 | 5673 | 7629 | 7517 | 6657 | 7469 | 360/40,460/40 Read#4 | |
| G | 159 | 159 | 191 | 163 | 177 | 173 | 7219 | 7261 | 6442 | 5981 | 5679 | 6446 | 360/40,460/40 Read#4 | |
| H | 174 | 162 | 168 | 170 | 176 | 177 | 11842 | 10457 | 7274 | 7606 | 4807 | 5150 | 360/40,460/40 Read#4 | |

| | 1 | 2 | 3 | 4 | 5 | 6 | 7 | 8 | 9 | 10 | 11 | 12 | | 20 |
|---|---|---|---|---|---|---|---|---|---|---|---|---|---|---|
| A | 169 | 167 | 156 | 168 | 173 | 186 | 188 | 231 | 9064 | 9220 | 11195 | 11974 | 360/40,460/40 Read#5 | |
| B | 326 | 161 | 154 | 153 | 186 | 182 | 187 | 182 | 5963 | 5636 | 5009 | 5612 | 360/40,460/40 Read#5 | |
| C | 171 | 162 | 167 | 162 | 291 | 172 | 201 | 188 | 7472 | 7873 | 10798 | 11493 | 360/40,460/40 Read#5 | |
| D | 203 | 160 | 189 | 164 | 174 | 173 | 300 | 184 | 6998 | 7043 | 6074 | 6246 | 360/40,460/40 Read#5 | |
| E | 167 | 162 | 165 | 166 | 172 | 168 | 207 | 200 | 10186 | 9884 | 11017 | 11921 | 360/40,460/40 Read#5 | |
| F | 164 | 158 | 180 | 165 | 167 | 198 | 6298 | 5933 | 8500 | 8491 | 7383 | 8294 | 360/40,460/40 Read#5 | |
| G | 158 | 158 | 191 | 161 | 176 | 172 | 8001 | 8107 | 7003 | 6515 | 5996 | 6779 | 360/40,460/40 Read#5 | |
| H | 174 | 160 | 168 | 169 | 176 | 178 | 14180 | 12373 | 8105 | 8455 | 4799 | 5152 | 360/40,460/40 Read#5 | |

| | 1 | 2 | 3 | 4 | 5 | 6 | 7 | 8 | 9 | 10 | 11 | 12 | | 25 |
|---|---|---|---|---|---|---|---|---|---|---|---|---|---|---|
| A | 170 | 166 | 156 | 166 | 175 | 186 | 186 | 230 | 10241 | 10483 | 12947 | 13938 | 360/40,460/40 Read#6 | |
| B | 328 | 160 | 152 | 154 | 185 | 182 | 186 | 182 | 6207 | 5846 | 5045 | 5653 | 360/40,460/40 Read#6 | |
| C | 172 | 162 | 167 | 163 | 291 | 172 | 202 | 187 | 8224 | 8816 | 12580 | 13390 | 360/40,460/40 Read#6 | |
| D | 205 | 158 | 190 | 164 | 174 | 173 | 302 | 182 | 7602 | 7598 | 6336 | 6594 | 360/40,460/40 Read#6 | |
| E | 165 | 160 | 165 | 165 | 171 | 169 | 207 | 198 | 11669 | 11360 | 12707 | 13820 | 360/40,460/40 Read#6 | |
| F | 164 | 155 | 182 | 162 | 166 | 198 | 6597 | 6213 | 9641 | 9584 | 8188 | 9231 | 360/40,460/40 Read#6 | |
| G | 158 | 159 | 189 | 162 | 175 | 172 | 8968 | 9032 | 7574 | 7079 | 6287 | 7147 | 360/40,460/40 Read#6 | |
| H | 174 | 160 | 169 | 168 | 175 | 180 | 16575 | 14294 | 8986 | 9351 | 4834 | 5185 | 360/40,460/40 | |

| | 1 | 2 | 3 | 4 | 5 | 6 | 7 | 8 | 9 | 10 | 11 | 12 | | 30 |
|---|---|---|---|---|---|---|---|---|---|---|---|---|---|---|
| A | 171 | 166 | 155 | 167 | 174 | 186 | 186 | 229 | 11528 | 11792 | 14734 | 15878 | 360/40,460/40 Read#7 | |
| B | 327 | 159 | 153 | 153 | 184 | 182 | 184 | 182 | 6550 | 6089 | 5111 | 5705 | 360/40,460/40 Read#7 | |
| C | 170 | 160 | 165 | 161 | 289 | 170 | 201 | 186 | 9048 | 9787 | 14456 | 15290 | 360/40,460/40 Read#7 | |
| D | 203 | 158 | 186 | 163 | 172 | 173 | 299 | 183 | 8174 | 8217 | 6647 | 6932 | 360/40,460/40 Read#7 | |
| E | 164 | 160 | 165 | 163 | 171 | 170 | 205 | 198 | 13240 | 12799 | 14418 | 15795 | 360/40,460/40 Read#7 | |
| F | 165 | 158 | 178 | 164 | 164 | 197 | 6910 | 6490 | 10940 | 10705 | 9031 | 10180 | 360/40,460/40 Read#7 | |
| G | 157 | 158 | 188 | 163 | 175 | 173 | 9866 | 10023 | 8243 | 7677 | 6669 | 7576 | 360/40,460/40 Read#7 | |
| H | 172 | 158 | 166 | 168 | 174 | 179 | 19021 | 16368 | 9889 | 10281 | 4868 | 5224 | 360/40,460/40 Read#7 | |

| | 1 | 2 | 3 | 4 | 5 | 6 | 7 | 8 | 9 | 10 | 11 | 12 | | 35 |
|---|---|---|---|---|---|---|---|---|---|---|---|---|---|---|
| A | 169 | 166 | 154 | 166 | 173 | 186 | 187 | 230 | 12788 | 13126 | 16591 | 17868 | 360/40,460/40 Read#8 | |
| B | 326 | 157 | 153 | 153 | 185 | 182 | 188 | 182 | 6815 | 6338 | 5145 | 5763 | 360/40,460/40 Read#8 | |
| C | 171 | 160 | 167 | 161 | 291 | 169 | 199 | 186 | 9882 | 10835 | 16355 | 16890 | 360/40,460/40 Read#8 | |
| D | 171 | 158 | 186 | 164 | 172 | 172 | 299 | 183 | 8855 | 8836 | 6945 | 7314 | 360/40,460/40 Read#8 | |
| E | 167 | 161 | 165 | 163 | 171 | 166 | 205 | 200 | 14815 | 14360 | 16254 | 17766 | 360/40,460/40 Read#8 | |
| F | 162 | 156 | 181 | 163 | 164 | 197 | 7223 | 6764 | 11916 | 11917 | 9926 | 11182 | 360/40,460/40 Read#8 | |
| G | 156 | 157 | 188 | 162 | 174 | 171 | 10796 | 11057 | 8865 | 8299 | 7028 | 7998 | 360/40,460/40 Read#8 | |
| H | 173 | 159 | 167 | 169 | 175 | 179 | 21401 | 18382 | 10839 | 11241 | 4908 | 5270 | 360/40,460/40 Read#8 | |

| | 1 | 2 | 3 | 4 | 5 | 6 | 7 | 8 | 9 | 10 | 11 | 12 | | 40 |
|---|---|---|---|---|---|---|---|---|---|---|---|---|---|---|
| A | 170 | 166 | 155 | 167 | 171 | 185 | 185 | 230 | 14091 | 14497 | 18377 | 19760 | 360/40,460/40 Read#9 | |
| B | 326 | 158 | 152 | 153 | 184 | 181 | 184 | 180 | 7106 | 6608 | 5186 | 5820 | 360/40,460/40 Read#9 | |
| C | 169 | 160 | 166 | 162 | 291 | 170 | 199 | 187 | 10749 | 11856 | 18293 | 18765 | 360/40,460/40 Read#9 | |
| D | 171 | 157 | 186 | 163 | 172 | 171 | 299 | 183 | 9457 | 9485 | 7261 | 7646 | 360/40,460/40 Read#9 | |
| E | 164 | 160 | 164 | 163 | 168 | 167 | 206 | 200 | 16372 | 15916 | 18099 | 19716 | 360/40,460/40 Read#9 | |
| F | 163 | 156 | 181 | 162 | 164 | 197 | 7539 | 7058 | 13345 | 13097 | 10796 | 12170 | 360/40,460/40 Read#9 | |
| G | 157 | 155 | 187 | 163 | 175 | 172 | 11736 | 12042 | 9504 | 8885 | 7402 | 8442 | 360/40,460/40 Read#9 | |
| H | 173 | 159 | 169 | 168 | 176 | 179 | 23804 | 20282 | 11768 | 12180 | 4963 | 5330 | 360/40,460/40 Read#9 | |

| | 1 | 2 | 3 | 4 | 5 | 6 | 7 | 8 | 9 | 10 | 11 | 12 | | 45 |
|---|---|---|---|---|---|---|---|---|---|---|---|---|---|---|
| A | 169 | 167 | 154 | 165 | 171 | 185 | 185 | 230 | 15378 | 15845 | 20227 | 21726 | 360/40,460/40 Read#10 | |
| B | 325 | 159 | 152 | 153 | 185 | 180 | 183 | 181 | 7361 | 6886 | 5250 | 5895 | 360/40,460/40 Read#10 | |
| C | 170 | 159 | 166 | 161 | 289 | 169 | 199 | 187 | 11635 | 12919 | 20200 | 20651 | 360/40,460/40 Read#10 | |
| D | 171 | 158 | 188 | 163 | 172 | 172 | 297 | 182 | 10204 | 10230 | 7590 | 8051 | 360/40,460/40 Read#10 | |
| E | 163 | 161 | 165 | 164 | 169 | 166 | 207 | 199 | 17988 | 17468 | 19965 | 21704 | 360/40,460/40 Read#10 | |
| F | 162 | 156 | 179 | 164 | 165 | 198 | 7853 | 7277 | 14323 | 14291 | 11710 | 13159 | 360/40,460/40 Read#10 | |
| G | 156 | 155 | 187 | 162 | 173 | 173 | 12676 | 13074 | 10183 | 9521 | 7807 | 8888 | 360/40,460/40 Read#10 | |
| H | 172 | 157 | 166 | 167 | 175 | 179 | 26147 | 22278 | 12736 | 13277 | 5027 | 5393 | 360/40,460/40 Read#10 | |

| | 1 | 2 | 3 | 4 | 5 | 6 | 7 | 8 | 9 | 10 | 11 | 12 | | 50 |
|---|---|---|---|---|---|---|---|---|---|---|---|---|---|---|
| A | 168 | 165 | 153 | 167 | 172 | 185 | 185 | 228 | 16705 | 17229 | 22037 | 23621 | 360/40,460/40 Read#11 | |
| B | 324 | 157 | 152 | 153 | 184 | 180 | 185 | 180 | 7680 | 7178 | 5288 | 5968 | 360/40,460/40 Read#11 | |
| C | 169 | 159 | 166 | 160 | 286 | 169 | 199 | 187 | 12532 | 14015 | 22116 | 22513 | 360/40,460/40 Read#11 | |
| D | 171 | 157 | 188 | 162 | 172 | 172 | 295 | 183 | 10881 | 10885 | 7955 | 8456 | 360/40,460/40 Read#11 | |
| E | 163 | 158 | 163 | 163 | 170 | 167 | 207 | 199 | 19524 | 19050 | 21806 | 23650 | 360/40,460/40 Read#11 | |
| F | 165 | 155 | 178 | 163 | 163 | 197 | 8201 | 7559 | 15543 | 15489 | 12615 | 14211 | 360/40,460/40 Read#11 | |
| G | 156 | 156 | 187 | 162 | 174 | 172 | 13616 | 14100 | 10844 | 10165 | 8213 | 9334 | 360/40,460/40 Read#11 | |
| H | 173 | 158 | 166 | 168 | 175 | 180 | 28390 | 24171 | 13663 | 14123 | 5075 | 5486 | 360/40,460/40 Read#11 | |

| | 1 | 2 | 3 | 4 | 5 | 6 | 7 | 8 | 9 | 10 | 11 | 12 | | 55 |
|---|---|---|---|---|---|---|---|---|---|---|---|---|---|---|
| A | 170 | 165 | 154 | 166 | 170 | 184 | 184 | 226 | 17964 | 18541 | 23805 | 25552 | 360/40,460/40 Read#12 | |
| B | 323 | 158 | 152 | 154 | 184 | 179 | 184 | 182 | 7986 | 7463 | 5350 | 6035 | 360/40,460/40 Read#12 | |
| C | 170 | 158 | 165 | 161 | 288 | 169 | 201 | 187 | 13418 | 15086 | 23941 | 24338 | 360/40,460/40 Read#12 | |
| D | 170 | 156 | 183 | 161 | 171 | 171 | 298 | 182 | 11568 | 11561 | 8308 | 8878 | 360/40,460/40 Read#12 | |
| E | 165 | 160 | 163 | 163 | 169 | 166 | 206 | 200 | 21116 | 20611 | 23611 | 25577 | 360/40,460/40 Read#12 | |
| F | 162 | 155 | 177 | 162 | 165 | 197 | 8530 | 7876 | 16768 | 16683 | 13573 | 15225 | 360/40,460/40 Read#12 | |

| | 1 | 2 | 3 | 4 | 5 | 6 | 7 | 8 | 9 | 10 | 11 | 12 | | 60 |
|---|---|---|---|---|---|---|---|---|---|---|---|---|---|---|
| A | 170 | 164 | 154 | 164 | 173 | 185 | 185 | 227 | 19269 | 19916 | 25584 | 27415 | 360/40,460/40 Read#13 | |
| B | 326 | 158 | 151 | 152 | 184 | 179 | 183 | 182 | 8388 | 7782 | 5399 | 6094 | 360/40,460/40 Read#13 | |
| C | 170 | 158 | 167 | 160 | 286 | 169 | 200 | 187 | 14326 | 16183 | 25855 | 26136 | 360/40,460/40 Read#13 | |
| D | 172 | 156 | 185 | 161 | 171 | 171 | 297 | 183 | 12259 | 12234 | 8711 | 9277 | 360/40,460/40 Read#13 | |
| E | 165 | 160 | 163 | 162 | 170 | 167 | 207 | 201 | 22677 | 22167 | 25424 | 27512 | 360/40,460/40 Read#13 | |
| F | 163 | 154 | 180 | 161 | 165 | 196 | 8884 | 8230 | 18021 | 17897 | 14507 | 16267 | 360/40,460/40 Read#13 | |
| G | 156 | 156 | 187 | 162 | 173 | 173 | 15546 | 16184 | 12227 | 11476 | 9060 | 10298 | 360/40,460/40 Read#13 | |
| H | 172 | 159 | 166 | 166 | 174 | 181 | 32907 | 28021 | 15573 | 16071 | 5202 | 5646 | 360/40,460/40 Read#13 | |

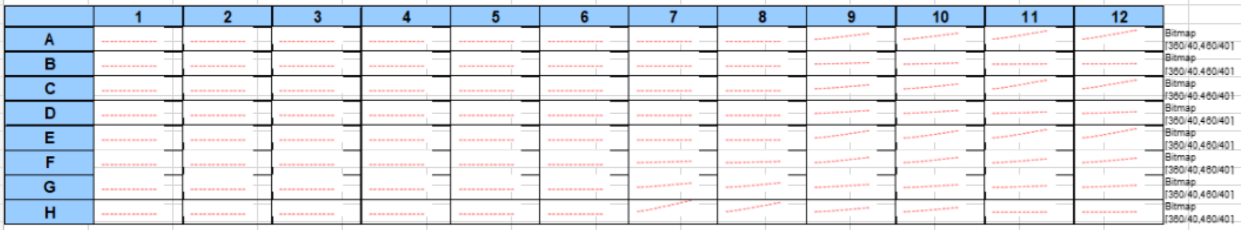

| | 0 | | 5 | | 10 | | 60 | |
|---|---|---|---|---|---|---|---|---|
| blanco | 4979 | 5354 | 4871 | 5231 | 4806 | 5158 | 5202 | 5646 |
| 2.2 | 5136 | 5657 | 5249 | 5823 | 5370 | 6116 | 9060 | 10298 |
| 2.3 | 5033 | 5561 | 5441 | 6052 | 6008 | 6710 | 14507 | 16267 |
| 2.5 | 5735 | 6058 | 6761 | 7149 | 7941 | 8518 | 25424 | 27512 |
| 2.7 | 5368 | 5380 | 5457 | 5489 | 5624 | 5706 | 8711 | 9277 |
| 2.10 | 5302 | 5608 | 6201 | 6722 | 7506 | 8089 | 25855 | 26136 |
| 2.11 | 5017 | 5539 | 4897 | 5577 | 4905 | 5549 | 5399 | 6094 |
| 2.12 | 5715 | 5893 | 6686 | 6990 | 7996 | 8460 | 25584 | 27415 |
| 2.19 | 5838 | 5861 | 6130 | 6172 | 6750 | 6848 | 15573 | 16071 |
| 2.20 | 5362 | 4957 | 5654 | 5196 | 5958 | 5561 | 12227 | 11476 |
| 7.2 | 5461 | 5414 | 5953 | 5937 | 6633 | 6661 | 18021 | 17897 |
| 7.3 | 5731 | 5499 | 6439 | 6244 | 7541 | 7218 | 22677 | 22167 |
| 7.5 | 5310 | 5484 | 5580 | 5702 | 5931 | 6044 | 12259 | 12234 |
| 7.7 | 5335 | 5077 | 5572 | 5588 | 6091 | 6273 | 14326 | 16183 |
| 7.10 | 5157 | 4946 | 5283 | 5007 | 5525 | 5192 | 8388 | 7782 |
| 7.11 | 5390 | 5367 | 6001 | 6087 | 6875 | 6900 | 19269 | 19916 |
| 7.12 | 6383 | 6335 | 7914 | 7430 | 9686 | 8950 | 32907 | 28021 |
| 7.14 | 5689 | 5638 | 5964 | 5959 | 6562 | 6526 | 15546 | 16184 |
| 7.16 | 5380 | 5166 | 5533 | 5286 | 5738 | 5446 | 8884 | 8230 |

| | promedio 5 | promedio 60 | 60-5 | UAF(mg*min) | |
|---|---|---|---|---|---|
| blanco | 5051 | 5424 | 373 | 135,636364 | |
| 2.2 | 5536 | 9679 | 4143 | 1506,54545 | |
| 2.3 | 5746,5 | 15387 | 9640,5 | 3505,63636 | control 2 |
| 2.5 | 6955 | 26468 | 19513 | | |
| 2.7 | 5473 | 8994 | 3521 | 1280,36364 | cont+glu 2 |
| 2.10 | 6461,5 | 25995,5 | 19534 | 7103,27273 | |
| 2.11 | 5237 | 5746,5 | 509,5 | | colitis 2 |
| 2.12 | 6838 | 26499,5 | 19661,5 | 7149,63636 | |
| 2.19 | 6151 | 15822 | 9671 | 3516,72727 | colitis+glu 2 |
| 2.20 | 5425 | 11851,5 | 6426,5 | 2336,90909 | |
| 7.2 | 5945 | 17959 | 12014 | 4368,72727 | |
| 7.3 | 6341,5 | 22422 | 16080,5 | | control 7 |
| 7.5 | 5641 | 12246,5 | 6605,5 | 2402 | |
| 7.7 | 5580 | 15254,5 | 9674,5 | 3518 | cont+glu 7 |
| 7.10 | 5145 | 8085 | 2940 | | |
| 7.11 | 6044 | 19592,5 | 13548,5 | 4926,72727 | colitis 7 |
| 7.12 | 7672 | 30464 | 22792 | 8288 | |
| 7.14 | 5961,5 | 15865 | 9903,5 | 3601,27273 | colitis+glu 7 |
| 7.16 | 5409,5 | 8557 | 3147,5 | 1144,54545 | |

| | |
|---|---|
| control 2 | 2506,09091 |
| cont+glu 2 | 1280,36364 |
| colitis 2 | 7126,45455 |
| colitis+glu 2 | 2926,81818 |
| control 7 | 4368,72727 |
| cont+glu 7 | 2960 |
| colitis 7 | 6607,36364 |
| colitis+glu 7 | 2372,90909 |

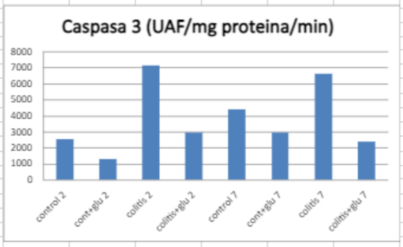


B28

| | A | B | C | D | E | F | G | H | I | J | K | L | M | N |
|---|---|---|---|---|---|---|---|---|---|---|---|---|---|---|
| 1 | caspasa 9 tiempo 0 | 1 | 2 | 3 | 4 | 5 | 6 | 7 | 8 | 9 | 10 | 11 | 12 | |
| 2 | A | 44868 | 44410 | 50481 | 40977 | 41806 | 41415 | 772 | 720 | 728 | 971 | 708 | 714 | 360/40,460/40 |
| 3 | B | 42274 | 38044 | 33402 | 37186 | 32935 | 35244 | 730 | 853 | 731 | 813 | 744 | 661 | 360/40,460/40 |
| 4 | C | 44118 | 34879 | 35268 | 35815 | 34964 | 38630 | 861 | 764 | 722 | 756 | 688 | 868 | 360/40,460/40 |
| 5 | D | 38501 | 37034 | 36728 | 38287 | 830 | 876 | 758 | 763 | 744 | 734 | 837 | 719 | 360/40,460/40 |
| 6 | E | 46210 | 38711 | 31703 | 32575 | 1656 | 854 | 878 | 766 | 803 | 752 | 715 | 782 | 360/40,460/40 |
| 7 | F | 37660 | 29484 | 36282 | 34289 | 833 | 847 | 813 | 1019 | 779 | 736 | 725 | 713 | 360/40,460/40 |
| 8 | G | 35950 | 30740 | 29884 | 28917 | 825 | 876 | 1070 | 740 | 733 | 693 | 730 | 724 | 360/40,460/40 |
| 9 | H | 728 | 722 | 34163 | 37050 | 843 | 868 | 1353 | 846 | 716 | 717 | 818 | 761 | 360/40,460/40 |
| 10 | | | | | | | | | | | | | | |
| 11 | | | | | | | | | | | | | | |
| 12 | | | | | | | | | | | | | | |
| 13 | caspasa 9 tiempo 45 | 1 | 2 | 3 | 4 | 5 | 6 | 7 | 8 | 9 | 10 | 11 | 12 | |
| 14 | A | 48874 | 49067 | 61344 | 51743 | OVRFLW | OVRFLW | 752 | 694 | 691 | 919 | 660 | 687 | 360/40,460/40 |
| 15 | B | 75119 | 65408 | 58196 | 61773 | 57121 | 62616 | 702 | 802 | 685 | 835 | 683 | 628 | 360/40,460/40 |
| 16 | C | OVRFLW | 70986 | 59786 | 84356 | 57820 | 78348 | 958 | 719 | 672 | 707 | 647 | 719 | 360/40,460/40 |
| 17 | D | OVRFLW | 88522 | 67277 | 90395 | 780 | 816 | 796 | 707 | 694 | 694 | 789 | 682 | 360/40,460/40 |
| 18 | E | 70958 | 61072 | 55984 | 62515 | 913 | 791 | 700 | 694 | 1056 | 689 | 671 | 730 | 360/40,460/40 |
| 19 | F | OVRFLW | 85655 | 84452 | 86141 | 751 | 794 | 737 | 692 | 707 | 687 | 678 | 675 | 360/40,460/40 |
| 20 | G | 49654 | 43133 | 57296 | 64151 | 771 | 820 | 1070 | 692 | 636 | 659 | 684 | 690 | 360/40,460/40 |
| 21 | H | 660 | 612 | 93471 | OVRFLW | 791 | 814 | 1222 | 793 | 814 | 669 | 764 | 719 | 360/40,460/40 |
| 22 | | | | | | | | | | | | | | |
| 23 | | | | | | | | | | | | | | |
| 24 | | | | | | | | | | | | | | |

**Figure 5A**

**PERK** Raw material

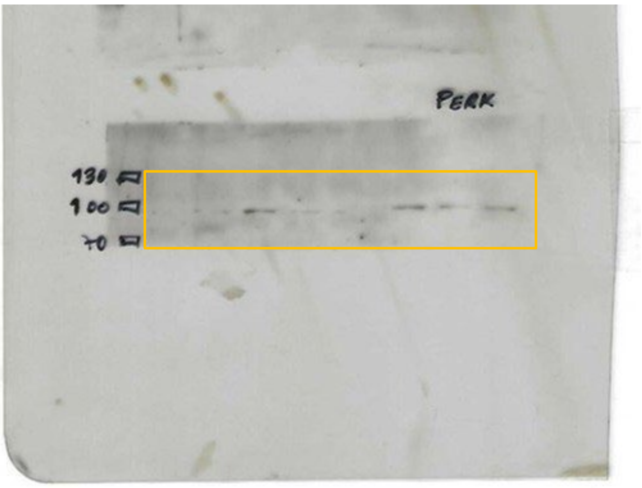


Published

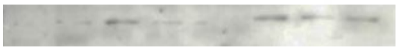

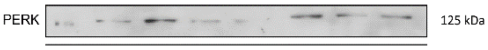


**ATF6** Raw material

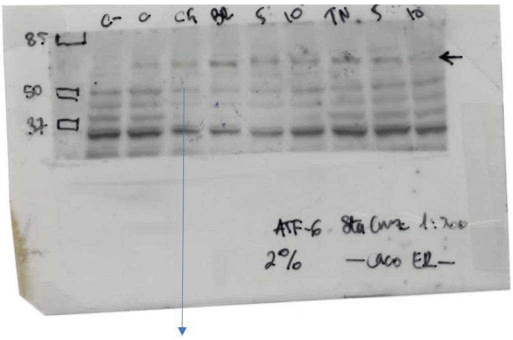


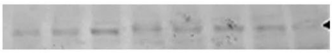

Published

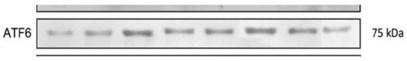

**BIP** Raw material

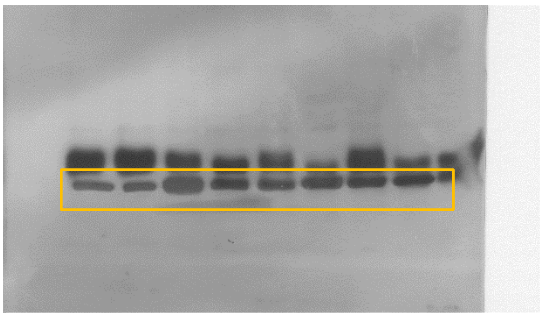

Published

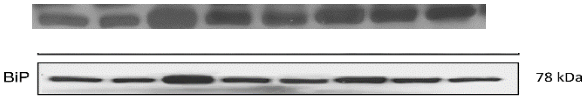


**Phospho-IRE1** Raw material

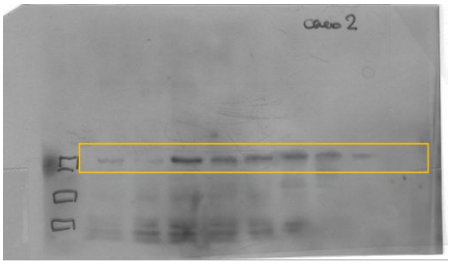

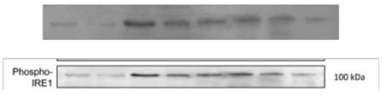


**Cleaved Caspas-3** Raw material

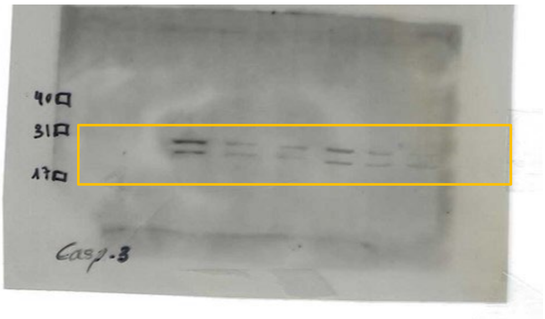

Published

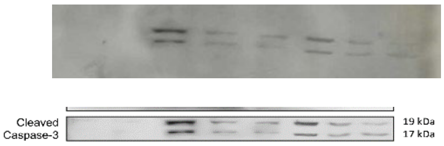

Supplement: S1 File — (PDF) [file pone.0297611.s001.pdf]
